# Genome Sequencing Identifies 13 Novel Candidate Risk Genes for Autism Spectrum Disorder in a Qatari Cohort

**DOI:** 10.3390/ijms252111551

**Published:** 2024-10-27

**Authors:** Afif Ben-Mahmoud, Vijay Gupta, Alice Abdelaleem, Richard Thompson, Abdi Aden, Hamdi Mbarek, Chadi Saad, Mohamed Tolefat, Fouad Alshaban, Lawrence W. Stanton, Hyung-Goo Kim

**Affiliations:** 1 Neurological Disorder Research Center, Qatar Biomedical Research Institute (QBRI), Hamad Bin Khalifa University (HBKU), Qatar Foundation, Doha 5825, Qatar; abenmahmoud@hbku.edu.qa (A.B.-M.); vgupta@hbku.edu.qa (V.G.); alicealeem@yahoo.com (A.A.); ithompson@hbku.edu.qa (R.T.); abaden@hbku.edu.qa (A.A.); falshaban@hbku.edu.qa (F.A.); 2 Medical Molecular Genetics Department, Human Genetics and Genome Research Institute, National Research Centre, Cairo 8854, Egypt; 3 Qatar Genome Program, Qatar Foundation Research, Development and Innovation, Qatar Foundation, Doha 5825, Qatar; hmbarek@qf.org.qa (H.M.); csaad@qf.org.qa (C.S.); 4 Shafallah Center for Children with Disabilities, Doha 2713, Qatar; mohamed.tolefat@shafallah.org.qa; 5 Department of Neurosurgery, Robert Wood Johnson Medical School, Rutgers, The State University of New Jersey, New Brunswick, NJ 08854, USA

**Keywords:** genome sequencing, autism spectrum disorder, Qatari cohort, novel genes, molecular pathways

## Abstract

Autism spectrum disorder (ASD) is a neurodevelopmental condition characterized by deficits in social communication, restricted interests, and repetitive behaviors. Despite considerable research efforts, the genetic complexity of ASD remains poorly understood, complicating diagnosis and treatment, especially in the Arab population, with its genetic diversity linked to migration, tribal structures, and high consanguinity. To address the scarcity of ASD genetic data in the Middle East, we conducted genome sequencing (GS) on 50 ASD subjects and their unaffected parents. Our analysis revealed 37 single-nucleotide variants from 36 candidate genes and over 200 CGG repeats in the *FMR1* gene in one subject. The identified variants were classified as uncertain, likely pathogenic, or pathogenic based on in-silico algorithms and ACMG criteria. Notably, 52% of the identified variants were homozygous, indicating a recessive genetic architecture to ASD in this population. This finding underscores the significant impact of high consanguinity within the Qatari population, which could be utilized in genetic counseling/screening program in Qatar. We also discovered single nucleotide variants in 13 novel genes not previously associated with ASD: *ARSF*, *BAHD1*, *CHST7*, *CUL2*, *FRMPD3*, *KCNC4*, *LFNG*, *RGS4*, *RNF133*, *SCRN2*, *SLC12A8*, *USP24*, and *ZNF746*. Our investigation categorized the candidate genes into seven groups, highlighting their roles in cognitive development, including the ubiquitin pathway, transcription factors, solute carriers, kinases, glutamate receptors, chromatin remodelers, and ion channels.

## 1. Introduction

The Arab populations from the Middle East and North Africa (MENA) region, traditionally underrepresented in whole-genome data studies, possess a remarkable genetic diversity that is intricately linked to their migration patterns, tribal structures, and high levels of consanguinity [1]. Consanguineous marriages are prevalent in the Middle East, including the Gulf countries, and range between 20% and 50% [2]. Qatar exhibits a consanguinity rate of around 54%, primarily in the form of first cousins’ marriages. Research indicates that consanguinity amplifies the occurrence of birth defects and genetic disorders [3,4,5,6]. The unique genomic variation patterns observed in MENA populations, particularly in the United Arab Emirates, Qatar, and Saudi Arabia, have provided critical insights into the genetic basis of both rare and common diseases [7,8,9]. Although Arab and Middle Eastern populations account for roughly 5% of the global population, autistic individuals from these regions represent a disproportionately small fraction of the total number of autism cases studied in genetic research. The underrepresentation of diverse populations in genetic research [10,11,12], could lead to unequal access to precision medicine, particularly for those bearing a heavier burden of disease [13]. In this context, the Qatari and Arab populations are vital due to the unique genetic diversity and cultural factors influencing the prevalence and manifestation of the disorder. These populations may have distinct risk genes linked to ASD, which can be overlooked in broader studies. Additionally, increased awareness and understanding of ASD in these communities can lead to improved diagnostic and support systems. The insights gained from this research can inform precision medicine approaches tailored to their specific needs, ultimately enhancing treatment outcomes and support for autistic individuals in these underrepresented groups [9,14,15]. Moreover, the findings could be utilized in genetic counseling and screening programs in Qatar, enhancing early diagnosis and intervention strategies. This focus on tailored counseling and screening could ultimately improve outcomes for individuals with ASD and their families.

Recent advances in genetic research demonstrate the value of larger-cohort genetic analyses in uncovering critical associations with human diseases. For instance, studies have identified significant genetic variants linked to various disorders by analyzing extensive datasets, highlighting the importance of including diverse populations [16]. These larger cohort studies improve our understanding of the genetic architecture of diseases and contribute to the development of personalized medicine approaches. In the context of ASD [17,18,19,20], leveraging insights from such comprehensive analyses is vital for identifying novel risk genes and elucidating their roles within the broader landscape of neurodevelopmental disorders (NDDs).

Autism spectrum disorder (ASD) is a neurodevelopmental disorder characterized by persistent challenges in social communication and interaction, along with restricted, repetitive patterns of behavior, interests, or activities. According to the Diagnostic and Statistical Manual of Mental Disorders, Fifth Edition (DSM-5), ASD encompasses a wide range of symptoms and functional abilities, affecting individuals differently in terms of speech and nonverbal communication, social interactions, and behavioral patterns [21]. The global prevalence of ASD in children is approximately 1%, with males being affected about four times more frequently than females [22,23]. Individuals with ASD may experience comorbid conditions such as epilepsy, intellectual disability (ID), and attention deficit hyperactivity disorder (ADHD) [22,24]. Our exploration of ASD acknowledges the frequent coexistence of ASD with ID, presenting challenges in identifying the underlying etiology of these conditions due to their heterogeneity [25,26].

In Qatar, one report estimates the overall prevalence of autism to be 1.14% [27]. Recent data from the U.S. Centers for Disease Control and Prevention (CDC), Centers for Disease Control and Prevention indicate that the prevalence of autism might be as high as 1 in 36 individuals (2.8%), which is significantly higher than earlier estimations.

Previously, we presented results from exome sequencing (ES) of six simplex autism trios from Qatar [28]. Here, we expand this genomic analysis using more comprehensive genome sequencing on a larger cohort of 50 family trios (mother, father, and affected child) with unknown genetic etiology to better understand the genetic prevalence, inheritance pattern, diagnostic rate, and genotype–phenotype correlations of autism candidate genes in cohorts of Mid-Eastern and African descent. We aim to identify the genetic variants in novel autism candidate genes and elucidate the biological pathways involved in this genetic disorder. This will contribute to the development of targeted therapies and tailored precision medicine approaches for ASD in underrepresented populations.

## 2. Results

### 2.1. Cohort Description

This investigation was endorsed by the Institutional Review Board (IRB) committee of the Qatar Biomedical Research Institute (QBRI), ensuring strict adherence to the ethical principles delineated in the Declaration of Helsinki. Written consent was obtained from the parents. A total of 150 participants were recruited primarily from the Shafallah Center for Persons with Disabilities (Doha, Qatar). The cohort consisted of 50 subjects, of which 40 were males and 10 were females. The cohort included the biological parents of each subject. In our study, 14 families reported a history of consanguinity in their marital background, which is significant as consanguinity increases the likelihood of inheriting recessive disorders. Each of these subjects was clinically diagnosed with ASD. To determine whether the children have ASD, the trained diagnostic team utilized the Autism Diagnostic Interview—Revised (ADI-R) and the Autism Diagnostic Observational Schedule (ADOS) [29].

During the recruitment phase, potential confounding variables such as environmental exposure, complications during childbirth, and other pregnancy associated factors were assessed for exclusion from this study. Language specification and tribe name or origin were not part of the inclusion criteria. Within the Shafallah cohort, the most frequently observed comorbidities were language and speech delays (60%), developmental delays (28%), intellectual disability (22%), and epilepsy (20%). Detailed age and clinical information for all 50 subjects are provided in Table 1. The participants were predominantly Qatari nationals, accounting for 66% (33/50) of the total cohort. The remaining 34% were expatriates from various countries, including Syria (10%), Egypt (4%), Yemen (4%), Sudan (4%), Saudi Arabia (2%), Algeria (2%), Jordan (2%), India (2%), Tunisia (2%), and Palestine (2%). The genetic diversity within our cohort, comprising participants from various nationalities, may influence the interpretation of our findings. Each nationality carries distinct genetic backgrounds, which could affect the risk factors associated with ASD. This heterogeneity complicates the identification of specific genetic variants linked to ASD, as variants may have different impacts across different ethnic groups. 

### 2.2. Sequencing and Analytical Pipeline Process

Genome sequencing was conducted on all participating children and their parents (n = 150 individuals), achieving an average read depth of 35X. Approximately 96% of bases were covered at a mean depth of 20X. The median number of single nucleotide variants (SNVs) present in each individual genome was 3.4 million, with 63,755 novel variants identified. We observed a transition to transversion (ti/tv) ratio of 2.05 and a heterozygote to non-ref homozygote (Het/Hom) ratio of 1.85, consistent with previous GS studies [30]. In our filtering process for single nucleotide variants (SNVs), we employed a minor allele frequency (MAF) threshold of <1% based on established guidelines from population databases such as the 1000 Genomes Project, gnomAD, and ExAC. This threshold was selected to focus our analysis on rare variants, which are more likely to have significant associations with ASD and reduce the potential for including common variants that may not contribute to the phenotype of interest. We obtained an average of 26,743 rare SNVs (95.3% heterozygous and 4.7% homozygous) and 4553 rare indels (87.8% heterozygous and 12.2% homozygous) per genome for further analysis.

Subsequently, we applied a CADD score threshold of >20 to prioritize variants predicted to have a deleterious effect on protein function. This score indicates the potential impact of variants on gene function, with higher scores suggesting greater likelihoods of pathogenicity. We also adhered to professional guidelines, particularly those from the American College of Medical Genetics and Genomics (ACMG), considering only variants classified as variants of unknown significance (VUSs), likely pathogenic, or pathogenic. By implementing these stringent filtering criteria, we aimed to enrich our dataset with potentially pathogenic variants that are relevant to the genetic underpinnings of ASD in our cohort. This method is consistent with previous genomic studies and enhances the robustness of our findings. 

Our initial dataset comprised 70 variants from 69 unique candidate genes. An expansion of more than 200 CGG repeats was found in the *FMR1* gene in subject 23. Detailed information about the selected variants and the applied criteria is presented in Table 2.

### 2.3. Refinement and Prioritization of ASD Candidate Genes

Genome sequencing of the Qatari population conducted by the Qatar Genome Project (QGP) identified an overlap of 55 M variants with dbSNP (build151), 46 M variants with gnomAD, and 244,233 variants with the GME database [9]. The latest version of the gnomAD database (3.1) contains data from only 158 Middle Eastern genomes [31]. In this context, we excluded variants possessing a high allele frequency (>1%) in the QGP databases. Additionally, we incorporated the tolerance for loss of the respective genes into our analysis, considering both the pLI (probability of being loss-of-function intolerant) and Z-scores. Specifically, for de novo variants, a stringent cut-off pLI score of > 0.9 was employed, especially in cases of nonsense/frameshift variants. For missense variants, a dual criterion of a Z-score exceeding +2.5, and a pLI score above 0.9 was required. Variants failing to meet these criteria were not shortlisted as strong candidate ASD risk genes.

Our final dataset unveiled 37 single nucleotide variants originating from 36 unique candidate genes, in addition to one expansion of over 200 CGG repeats in the *FMR1* gene in one subject. The single variants comprised thirty-one missense (84%), three frameshift (8.1%), one acceptor splice site (2.7%), one in-frame GCA deletion (2.7%), and one start loss (2.7%). They were classified as 19 homozygous (52%), 10 X-linked (28%), 6 de novo (17%), and one compound heterozygous (2.8%) variants.

The fact that more than 50% of variants are found in a homozygous state indicates a substantial contribution of recessive genetic mechanisms to ASD within this population. Additionally, the presence of de novo variants (17%) is noteworthy, as these mutations are often implicated in developmental disorders and may signify a strong genetic contribution to ASD risk. The identification of X-linked variants (28%) further highlights the role of sex-linked genes in ASD, suggesting a relationship to the observed male predominance of the disorder. Collectively, this variant profile provides important insights into the genetic mechanisms underlying ASD in our cohort (For a more detailed account, please refer to Table 2).

### 2.4. Identification of 13 Novel ASD-Risk Genes

Genes linked to mental and developmental diseases may also be associated with ASD [32,33]. In this study, we used the genomic approach of “parent–offspring trio genome sequencing” to analyze 50 subjects with ASD. Our findings identified 37 genes as having a high likelihood of being causative for ASD. This determination was based on several stringent criteria including low prevalence in the population, high-quality sequencing coverage, high CADD score, significant probability of being loss-of-function intolerant (pLI) and Z-scores, and alignment with the American College of Medical Genetics and Genomics (ACMG) guidelines.

Among these, 24 genes had variants previously associated with autism and/or other NDDs (Table S1). Additionally, we identified 13 novel candidate genes (*ARSF*, *BAHD1*, *CHST7*, *CUL2*, *FRMPD3*, *KCNC4*, *LFNG*, *RGS4*, *RNF133*, *SCRN2*, *SLC12A8*, *USP24*, and *ZNF746*) with limited information or whose specific mechanisms linking their variants to the autism phenotype are not yet understood (Table S1). These genes participate in various biological processes, including chromatin or DNA binding activity, protein–protein or protein–DNA interactions, and GTPase-activating proteins, underscoring the multifaceted genetic origins of autism.

In a bid to further substantiate our findings, we juxtaposed our newly identified gene list, comprising 37 genes, with the established neurodevelopmental disorder (NDD)/ASD gene list from Genomics England NDD/Autism panel genes (https://panelapp.genomicsengland.co.uk/panels/285/), (accessed on 1 July 2024), and the Simons Foundation Autism Research Initiative (SFARI) genes list (https://gene.sfari.org/database/human-gene/) (accessed on 1 July 2024),. Genes from our list of 37 that were also present in these established databases were denoted as present (“P”) or not present (“NP”) in Table S1. Interestingly, this comparative analysis revealed that 11 out of the 37 genes were shared with the SFARI gene list, while 17 genes were common to the Genomics England NDD/Autism panel genes. This overlap further underscores the potential significance of these genes in the etiology of ASD, thereby bolstering the evidence supporting their involvement in this disorder.

### 2.5. Sporadic Variants Reported in Our ASD Candidate Genes

Many of the candidate genes listed in Table 2 have sporadic variants identified in neurodevelopmental patients through next-generation sequencing. For instance, several missense variants in *DNAH3* (Dynein Axonemal Heavy Chain 3, MIM# 603334) have been reported in individuals with ASD [17,20]. Furthermore, five de novo missense variants in *DNAH3* have been documented in developmental disorders [34], along with two missense variants in ID [35,36]. Another gene, *OBSL1* (Obscurin-like 1, MIM# 610991), has one missense and two silent variants identified in individuals with ASD [17,20], as well as seven silent and two missense variants identified in developmental disorders [20,37]. A total of 44 sporadic variants, including missense, silent, nonsense, and splice alterations, have been reported in *CSMD1* (CUB and Sushi multiple domains 1, MIM# 608397) in individuals with developmental disorders and autism [17,20,34,37,38,39,40], suggesting that *CSMD1* is likely an autism disorder gene. Lastly, one missense and one frameshift variant in *E2F8* (E2F transcription factor 8, MIM# 612047) have been reported in individuals with ASD [17,20,41], along with one missense alteration in a developmental disorder [20,37] (Table S1).

## 3. Discussion

Since this study utilized genome sequencing (GS), it is essential to emphasize the key advantages of GS compared to exome sequencing (ES). One significant benefit of GS is its ability to identify intronic variants that ES cannot detect, thereby providing a more comprehensive view of the genome. This inclusion of non-coding regions allows for a better understanding of genetic variations that may influence gene expression and contribute to ASD. However, interpreting intronic variants poses challenges, primarily due to the limited availability of curated databases and in-silico tools designed to assess their pathogenicity and impact on gene function. This limitation underscores the need for ongoing research to develop robust methodologies for evaluating the effects of intronic and intragenic variants in both ASD and other neurodevelopmental disorders, ultimately enhancing our understanding of their roles in disease mechanisms. It was evident from the list of candidate SNVs in our Table 2 where we identified six intronic variants in six subjects but none of the variants made to the final category of 37 candidate SNVs due to the lack of supporting evidence in the literature and low priority/ranking in our bioinformatics analysis.

In our exploration of the genetic basis of ASD, several pathways have emerged as critical areas of focus due to their potential roles in the development and manifestation of these conditions. Firstly, the ubiquitin pathway, a crucial regulator of almost all cellular processes, has been implicated in the pathogenesis of ASD. The dysregulation of this pathway could provide insights into new therapeutic interventions. Secondly, transcription factors, which play pivotal roles in regulating gene expression, have been associated with ASD. Thirdly, the solute carrier (SLC) family of proteins, responsible for transporting a wide variety of solutes across biological membranes, are of great interest due to their links to ASD. Their roles in neurotransmission, nutrient uptake, and waste removal make them crucial in maintaining cellular homeostasis. Finally, phosphatases and kinases, key players in cellular signaling, are known to be involved in many neurodevelopmental disorders, including ASD. These pathways and their interactions could provide valuable insights into the underlying mechanisms of ASD, paving the way for improved clinical stratification and targeted therapeutic development (Table S1).

### 3.1. Identification of Ubiquitin Pathway-Associated Genes as Potential ASD Causative Factors in Five Subjects in Our Cohort

Several studies have indicated that the ubiquitin system modulates critical neural processes, such as axonal initiation, development, dendritic maturation, and synaptic pruning, which are often disrupted in ID and ASD [42]. Recent research has shown that mutations in genes encoding ubiquitin ligases and deubiquitinating enzymes can disrupt synaptic plasticity and neuronal signaling, thereby contributing to neurodevelopmental phenotypes [43]. Additionally, several studies demonstrated how dysregulation of the ubiquitin–proteasome system impacts neurogenesis and could lead to neurodevelopmental abnormalities associated with ASD [44,45,46]. These findings suggest that perturbation in the ubiquitin pathway could be a common mechanism underlying various neurodevelopmental disorders, including ASD. In our cohort, five candidate genes associated with the ubiquitin pathway were identified, potentially elucidating the autistic features observed in our subjects. *CUL2* (CULLIN2, MIM# 603135), identified in subject 2, is a scaffold protein in the Cullin-RING E3 ubiquitin ligase complexes (CRLs). These complexes are essential for targeting proteins for ubiquitination and subsequent degradation by the proteasome. Specifically, CUL2 forms a VCB-CUL2 complex with the Von Hippel–Lindau (VHL) protein, Elongin B and C, and RBX1. This complex has been shown to function as a ubiquitin ligase. Missense, nonsense, and splice variants in *CUL2* have been identified in patients with ASD [20,47] and developmental disorders [37]. *USP24* (MIM# 610569), a ubiquitin-specific peptidase gene identified in subject 10, belongs to a large family of cysteine proteases and functions as deubiquitinating enzymes [48]. This gene has been implicated in Parkinson’s disease [49] and developmental disorders [34].

Notably, a de novo missense variant c.3497C>T (NM_015306.3); p.A1166V (NP_056121.2) has been reported in five unrelated subjects with ASD [34,38,41,50,51]. *USP9X* (MIM# 300072), an additional ubiquitin-specific X-linked gene identified in subject 38, has been reported to have various nonsense and missense variants in NDDs [34,52,53,54,55]. One synonymous variant and multiple pathogenic missense variants in *USP9X* are identified with autism [34], and ASD [34,38,41,55,56,57]. *CCDC88C* (coiled-coil domain containing 88C, MIM# 611204), identified in subject 41, encodes a ubiquitously expressed coiled-coil domain-containing protein that interacts with the disheveled protein and negatively regulates the Wnt signaling pathway. CCDC88C interacts with UBE2H (ubiquitin conjugating enzyme E2 H) [58], which is involved in the ubiquitin pathway. This gene has been implicated in hydrocephalus [59,60,61,62,63] and various missense variants in autism and ASD [38,40,50,51,64,65,66].

Finally, *RNF133* (MIM# 620556), a ring finger protein gene discovered in subject 8, is predicted to facilitate ubiquitin protein ligase activity and ubiquitination. This gene has been documented with developmental disorders [20,37] and ASD [17,20,34].

Targeting ubiquitin signaling, crucial for neuronal function, may offer transformative treatments for ASD. Enhancing the clearance of toxic protein aggregates and addressing neuronal vulnerabilities could slow or halt neurodevelopmental progression. Additionally, pharmacological strategies aimed at this pathway may complement emerging therapies like stem cell transplantation, potentially reversing neurodevelopmental impairments. Recent advancements in liquid biopsy techniques for neuroblastoma highlight the potential of non-invasive approaches to monitor brain diseases in children [67]. This raises the question of whether similar methodologies could be applied to ASD. Although ASD primarily involves neurodevelopmental changes, liquid biopsies may help identify biomarkers related to its genetic and epigenetic landscape, offering insights into neuroinflammation and neuronal damage. As this field evolves, exploring liquid biopsy applications could lead to early diagnosis and personalized treatment strategies for ASD [67].

### 3.2. Potential Role of Transcription Factor-Associated Genes in ASD: A Study on Four Subjects from Our Cohort

Neural development requires the precise orchestration of various regulatory cascades to create a functional network. Dysregulation of transcription and translation during neurodevelopment can impair neurogenesis, neuronal migration, differentiation, and synaptic function [68]. Transcription factors play a critical role in these processes by controlling gene expression and chromatin remodeling. Recent studies have identified several transcription factors as pivotal in the pathogenesis of ASD [69,70,71,72]. In our cohort, two candidate genes that are intrinsically linked to transcription factor pathways were identified. These genes—*USF3* (Upstream Transcription Factor Family Member 3, MIM# 617568), and *TAF7L* (TATA-box Binding Protein Associated Factor 7 like, MIM# 300314)—may potentially elucidate the phenotypic manifestations of ASD observed in the subjects. *USF3*, identified in subject 16, has previously been reported in childhood disintegrative disorder [73,74] and schizophrenia [75]. Moreover, it has been proposed as a candidate gene for ASD in unrelated subjects [41,51].

*TAF7L*, a constituent of the DNA-binding general transcription factor complex TFIID, was identified in subject 34. Pathogenic variants in this X-linked gene have been documented in a developmental disorder [54] and schizophrenia [76]. Additionally, zinc/RING-finger proteins, such as MAGEC1, found in subject 6, RNF133 and ZNF746, identified in subject 8, are instrumental in transcriptional regulation. These proteins, typified by a zinc-or RING-finger domain, possess the ability to interact with DNA, RNA, proteins, and small molecules [77,78]. This interaction, particularly with DNA, enables them to function as transcription factors, thereby modulating the expression of specific genes by either enhancing or suppressing the transcription process [79,80]. Considering their crucial role in transcriptional regulation, genetic alterations in these zinc/RING finger proteins could conceivably result in dysregulated gene expression. This dysregulation could subsequently precipitate a myriad of disorders, including NDDs such as ASD. Sporadic variants in *MAGEC1* (MIM# 300223) are well documented in patients with DD [20,37,54], schizophrenia [76], and, notably, ASD [20,34,38]. Sporadic missense variants in *RNF133* (MIM# 620556) have been reported in patients with DD [20,37] and ASD [17], while missense variants in *ZNF746* (MIM# 613914) are reported in patients with Parkinson’s disease [81], and DD [20,37].

### 3.3. Potential Contribution of Solute Carrier (SLC) Genes to ASD: Findings from Two Subjects in Our Cohort

Solute carrier (SLC) genes encode membrane transporters that are instrumental in the absorption and efflux of diverse chemicals across cellular membranes. Crucially, by modulating the movement of neurotransmitters, SLC genes play a pivotal role in the pathogenesis of NDDs. Mutations in these genes have been linked to a spectrum of neurological conditions, including epilepsy, ASD, and intellectual impairment. The significant contribution of SLC genes to the etiology and progression of brain disorders, including ASD, has been well-documented in the literature. In our cohort, we identified *SLC12A8* (Solute Carrier Family 12 Member 8, MIM# 611316), a nicotinamide mononucleotide transporter, in subject 31 as a potential contributor to the observed autistic features. Previous studies have proposed *SLC12A8* as a candidate gene in ASD research [17,20]. Another potential candidate gene, *SLC25A42* (MIM# 610823), identified in subject 47, belongs to the solute carrier family 25. This gene has been reported in various NDDs, including epileptic encephalopathy [82,83], and ASD [17,20].

### 3.4. Potential Contribution of a Kinase-Associated Gene to ASD: A Case Study from Our Cohort

Emerging research underscores the pivotal role of genes involved in protein kinase pathway in the etiology of NDDs, including ASD [84,85,86,87]. Within our cohort, one candidate gene associated with this pathway was identified, potentially illuminating the observed autistic features in the subject. Specifically, *CNKSR2* gene (Connector Enhancer of Kinase Suppressor of Ras 2, MIM# 300724), identified in subject 44, is an X-linked gene intricately involved in protein kinase pathways. Variations in this gene have been reported in a spectrum of developmental and NDDs, non-syndromic ID, and ASD [34,54,88,89,90,91,92,93,94].

### 3.5. Implication of Glutamate Receptor Genes GRIA2 and GRIN3B in ASD: Findings from Subjects 17 and 50

Glutamate receptors play a pivotal role in ASD. These receptors, which are integral components of the excitatory neuronal signaling pathway, are critical for synaptic plasticity, a key process in learning and memory. Recent research has highlighted the potential contribution of dysregulated glutamate signaling to the pathophysiology of ASD [95]. In our cohort, we identified variants in two genes encoding glutamate receptors: *GRIN3B* and *GRIA2*. *GRIN3B* (Glutamate Ionotropic Receptor NMDA Type Subunit 3B, MIM# 606651), encodes a subunit of the NMDA receptor, a type of ionotropic glutamate receptor, while *GRIA2* (Glutamate Ionotropic Receptor AMPA Type Subunit 2, MIM# 138247) encodes a subunit of the AMPA receptor. Both receptors are crucial for glutamatergic neurotransmission. Variants in these genes could potentially disrupt the normal functioning of glutamate signaling, thereby contributing to the neurodevelopmental abnormalities observed in ASD. The gene *GRIA2*, a known entity in the field of neurogenetics, has been previously documented with a spectrum of NDDs. These include ID [96], epilepsy [97], cognitive impairment [98], seizure [96], schizophrenia [99], NDD with language impairment and behavioral abnormalities [100], and NDD with refractory epilepsy [101], in addition to ASD [17,19,20,34,40,41,51]. Comparably, the *GRIN3B* gene, has been implicated in schizophrenia [102,103,104], general developmental disorders [20,37], and, notably, ASD [17,20,34,104].

### 3.6. Identification of Key Genes in Chromatin Remodeling as Potential Contributors to ASD in Subjects 3 and 25

Chromatin remodeling enzymes play a crucial role in regulating gene expression by modifying the structure of chromatin, which is essential for proper neurodevelopment. Dysregulation of these enzymes has been linked to various NDDs, including ASD and ID, highlighting their importance in brain development and function [105]. Histone lysine methyltransferases (KMTs) and demethylases (KDMs), known as “writers” and “erasers” in chromatin remodeling, play pivotal roles in neurodevelopment by adding and removing methyl groups from histones to regulate gene expression. For example, recessive histone lysine methylation defects caused by homozygous or compound heterozygous variants in *KDM5B* (lysine demethylase 5B, MIM# 605393) result in a recognizable neuropsychiatric syndrome characterized by developmental delay, ID, facial dysmorphism, and camptodactyly [106]. Genetic variants in another lysine demethylase, *KDM6B* (lysine demethylase 6B, MIM# 611577), are also associated with Stolerman syndrome characterized by DD, ID, and facial dysmorphism [107,108]. Our ASD candidate gene, as a KDM, is *KDM2A* (lysine demethylase 2A, MIM# 605657), found as a de novo missense variant in subject 25. KDM2A, a Jumonji C domain-containing demethylase, specifically demethylates histone H3 at lysine 36 (H3K36), playing a crucial role in regulating gene expression and chromatin structure. A dozen missense, silent, intronic, and frameshift variants in *KDM2A* have been documented in individuals diagnosed with ASD [20,34,38,41], and schizophrenia [109]. Histone deacetylases (HDACs) are crucial in chromatin remodeling by removing acetyl groups from histone tails. *BAHD1* (Bromo-Adjacent Homology Domain-Containing 1, MIM# 613880) acts as a transcriptional repressor. It is a core component of an HDAC1/2-associated complex, as well as other chromatin-associated complexes like the NuRD complex. These interactions facilitate histone deacetylation, leading to chromatin condensation and transcriptional repression [110]. Our Qatari individual, subject 3, with ASD and language delay, carried a homozygous missense variant in this gene, while one silent and eight missense heterozygous variants reported sporadically in *BAHD1* in ASD individuals were heterozygous [17,20,37].

### 3.7. Potential Contribution of the Voltage-Gated Ion Channel Genes KCNK9 and KCNC4 to ASD in Subjects 18 and 49

Emerging data from genome and exome sequencing studies have identified seven ASD-risk genes associated with potassium channels, which play a key role in regulating neuronal excitability [111]. *KCNC4* (potassium voltage-gated channel subfamily C member 4, MIM# 176265) forms a potassium-selective channel to mediate the voltage dependent potassium ion permeability of excitable membranes and is situated at 1p13.3. Through comparative genomic mapping, we previously identified *KCNC4* as one of eleven potential autosomal dominant candidate genes for syndromic NDDs that may have been dysregulated by the position effect of heterozygous CNVs at 1p13.3 [112]. Heterozygous missense variants in this gene have been found in individuals with ataxia and dystonia [113], as well as bipolar disorder [114]. Furthermore, a truncated KCNC4 protein due to a heterozygous frameshift variant has been identified in a patient with a developmental disorder [34,90]. In contrast to these findings, we identified a homozygous missense variant in *KCNC4* in a male subject of Saudi Arabian descent diagnosed with ASD. In addition to *KCNC4*, we also identified a de novo missense variant in another potassium channel gene, *KCNK9* (Potassium two pore domain channel subfamily K member 9, MIM# 605874), in our cohort. The two-pore-domain potassium channel (K2P channel) encoded by this gene is responsible for regulating the flow of potassium ions through cell membranes, playing crucial roles in maintaining the resting membrane potential and shaping the action potential in neurons and other excitable cells. *KCNK9* has been implicated in a diverse array of NDDs and syndromes. Notably, it has been associated with Birk–Barel intellectual disability–dysmorphism syndrome, a maternally transmitted imprinting condition characterized by ID, hypotonia, and unique facial dysmorphism [115,116,117,118,119]. Furthermore, variants in *KCNK9* have been identified in individuals with ASD [120,121].

### 3.8. The Intersection of Autism Spectrum Disorder, Depression, Sleep Disorders, and Therapies

In the context of our genome sequencing findings, identifying 13 novel candidate risk genes for ASD, exploring interactions between ASD and mental health comorbidities like depression, takes on added significance. Individuals with ASD often face elevated levels of anxiety and depression [122], possibly influenced by genetic factors elucidated through our research. Additionally, the prevalence of sleep disturbances in ASD individuals, worsened by disrupted sleep patterns [123], highlights the importance of investigating the genetic basis of these issues in the context of our identified genes. Understanding the genetic underpinnings of these intersecting conditions could pave the way for personalized treatments and non-traditional therapies [124] to address the complex needs of individuals with ASD and mental health comorbidities.

## 4. Materials and Methods

### 4.1. Sampling Procedures, DNA Isolation, and Fragile X Screening

This study was approved by the Institutional Review Board under project QBRI-2010-002 titled “Study of Genetic and Environmental Etiologic Factors in Autism”. The bio-samples collected for this study mainly included blood and saliva from affected individuals and their parents. The samples were collected at the Shafallah center or collaborating hospitals in Doha, Qatar, using anti-coagulant-treated tubes containing ethylenediamine tetra acetic acid (EDTA) with lavender tops for DNA and RNA extraction. Genomic DNA was extracted from peripheral blood leukocytes using the Flexigene DNA extraction kit protocols (Qiagen, Hilden, Germany). The concentration of DNA samples was measured using Nanodrop Spectrophotometer 1000 (ND-1000; Thermo Fisher Scientific, Waltham, MA, USA) and Qubit (Thermo Fisher Scientific, USA) based methods as a part of quality control to ensure that no RNA impurities were included. Fragile X screening was performed on all subjects. The DNA samples were relabeled, diluted, and aliquoted at 2 ug each in new barcoded vials and were sent to the Qatar Genome Program for genome sequencing. Written informed consent was obtained from all subjects involved in this study.

### 4.2. Library Construction and Genome Sequencing

The genome sequencing for this study was performed as previously described [9]. Quality control procedures were used to ensure proper sample identification and genetic compatibility. The library construction and sequencing were carried out at the Sidra Clinical Genomics Laboratory Sequencing Facility. The Agilent SureSelectXT kit (Twist Bioscience HQ, South San Francisco, CA, USA) was used for library preparation following the manufacturer’s protocol. The process began by mechanically fragmenting 200 ng of gDNA using the Covaris E220 ultrasonicator system, followed by purification of the sheared DNA with AMPure XP magnetic beads. End-repair, adenylation, and ligation to the SureSelect DNA adapter were performed on the purified DNA. The ligated DNA was repurified and amplified by PCR. The library DNA was then hybridized to specific biotin probes, captured by streptavidin beads, and amplified by PCR using a specific index. The quality of the library was checked using Agilent 2100 Bioanalyzer system and quantified using the Qubit system. The libraries that passed quality control were pooled and sequenced on an Illumina HiSeq 4000 at a minimum of 50 million paired-end reads (2 × 150 bp) per sample.

### 4.3. GS and Variant Calling Process

**Figure 1:**
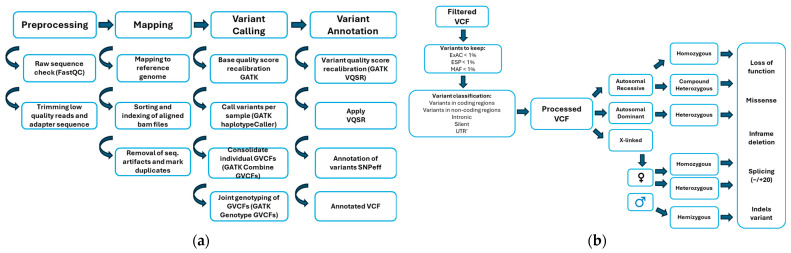
(**a**) Process of raw data quality control, sequence mapping, and the variant identification and filtering pipeline. The protocol adheres to GATK’s recommended best practices for discovering germline short variants. The pipeline begins with raw sequencing data undergoing quality control using FastQC, which assesses base quality, GC content, and adapter contamination. Low-quality reads are trimmed, and adapters are removed using standard tools. The cleaned reads are aligned to the reference human genome (GRCh38), followed by duplicate read marking and base quality score recalibration. Germline variant calling is performed using GATK HaplotypeCaller, which generates individual sample GVCF files. These GVCFs are then combined using GATK CombineGVCFs to create a cohort-wide variant call set. To ensure variant quality, GATK VQSR (Variant Quality Score Recalibration) is applied, which assigns confidence levels to the variant calls and filters out low-quality variants. (**b**) Variant filtering and functional annotation pipeline. The method for pinpointing variants that are likely to impact function is shown. After the initial variant calling and annotation, the filtered VCF undergoes a multi-step filtration process. Variants are first filtered based on their minor allele frequency (MAF) across population databases, excluding common variants with MAF >1%. This includes data from ExAC (Exome Aggregation Consortium), ESP (Exome Sequencing Project), and other population resources, applying a threshold of MAF < 1%. Next, variants are classified based on their genomic location, including coding and non-coding regions, such as intronic, silent (synonymous), UTR (untranslated regions), and exonic regions. Functional variants in coding regions are prioritized, particularly those affecting protein sequences. Variants are also categorized based on their impact, including loss-of-function (e.g., stop-gain, frameshift), missense, in-frame deletions, and variants affecting splicing and indels (insertions or deletions).

The Clinical Genomic Laboratory (CGL) provides the data in Fastq format. Initial quality control measures are executed on the Fastq files using FastQC (v0.11.2), which calculates quality metrics to ensure the optimal quality of raw reads. The reads are subsequently trimmed and aligned to the hs37d5 reference genome via the application of bwa.kit (v0.7.12), resulting in the generation of a BAM file. Quality control is then executed on the mapped reads (BAM files) using Picard (v1.117) [CollectWgsMetrics] to evaluate sample coverage. Variant calling adheres to the GATK 3.4 best practices, which includes indel realignment and base recalibration (BQSR) performed on the initial BAM file (Figure 1). The HaplotypeCaller is then applied to each sample to generate an intermediate genomic gVCF. Joint Genotyping is executed using all generated gVCF files simultaneously. Initially, GenomicsDB is run to combine the different samples by regions. Subsequently, GenotypeGVCFs is run on each region, SNP/Indel recalibration (VQSR) is applied, and all regions are merged. Annotation is performed using SnpEff/SnpSift (v4.3t). In the annotation of the multisamples VCF file, the following databases are utilized within SnpEff/SnpSift: dbSNP build 151, ClinVar 2019-02-11, dbNSFP v2.9, GWAS catalog, and msigDBdb v5.0.

### 4.4. SFARI Genes Database and Known ASD/NDD Panel Genes: An Annotation Approach for Additional Evidence

To find additional evidence supporting our findings, we undertook an annotation of the identified genes utilizing two comprehensive resources: the SFARI (Simons Foundation Autism Research Initiative) gene database and the Genomics England NDD/Autism panel genes. The SFARI gene database serves as an extensive repository of genes that have been potentially implicated in ASD. Conversely, the Genomics England NDD/Autism panel comprises genes associated with a range of conditions, including NDDs, neurology, ID, as well as autism. We conducted a comparative analysis by cross-referencing our newly identified gene list with these established in Genomics England NDD/ASD and SFARI gene lists. Genes that were identified as common to these lists are denoted as present (“P”) or not present (“NP”) in Table S1.

## 5. Conclusions

This study is one of the earliest studies identifying causative genetic variants in a Qatari cohort. Here, we have identified 37 distinct candidate genes consisting of de novo, X-linked (hemizygous), and homozygous (missense, frameshift, nonsense, insertion/deletion, and splice variants) variants belonging to known and/or novel genes associated with ASD. Multiple individuals displayed more than one candidate variant, raising the possibility of digenic models. Notably, these variants are absent in the gnomAD/publicly available population databases and exhibit extremely low frequencies in the local control population dataset. Furthermore, the presence of multiple de novo mutations across trios underscores the significant contribution of de novo mutations to the genetic etiology of ASD. Interestingly, 52% of the variations were found to be homozygous, suggesting that a significant fraction of the etiology of ASD in this population may be due to a recessive genetic architecture, highlighting the increased levels of consanguinity in this region. Further investigations of recessive alleles in these families can give important insight into genetic counseling to at-risk families. Moreover, effects of consanguinity on allele frequency and genetic diversity can help in public health planning and genetic screening programs in Qatar.

On the other hand, our study identifies several novel genetic variants associated with ASD, including genes not previously linked to the disorder, which could provide new insights into the genetic underpinnings of ASD. The identification of rare, potentially pathogenic variants, especially in a cohort with a high prevalence of consanguinity, underscores the importance of considering population-specific genetic factors in ASD research. These findings may help inform the development of future diagnostic tools that utilize targeted gene panels for early and accurate diagnosis.

Furthermore, the discovery of novel genes associated with ASD could open new avenues for therapeutic strategies, allowing for the exploration of personalized treatments aimed at specific genetic profiles. Continued research into these genetic pathways may lead to more precise and effective interventions for ASD.

## Web Resources

OMIM: https://www.omim.org/ (accessed on 1 July 2024).
HGMD: https://my.qiagendigitalinsights.com/bbp/view/hgmd/pro/all.php (accessed on 1 July 2024).
HGVS Nomenclature: http://varnomen.hgvs.org/ (accessed on 1 July 2024).
HUGO Gene Nomenclature Committee (HGNC): http://www.genenames.org (accessed on 1 July 2024).
Genomics England NDD/autism panel genes: https://panelapp.genomicsengland.co.uk/panels/285/ (accessed on 1 July 2024).
SFARI (Simons Foundation Autism Research Initiative): https://gene.sfari.org/database/human-gene/ (accessed on 1 July 2024).

## Figures and Tables

**Table 1:** Nationality and detailed clinical information of the 50 subjects diagnosed with ASD in Qatar. All data presented are based on the latest available examination.

Subject ID	Subject 1	Subject 2	Subject 3	Subject 4	Subject 5	Subject 6	Subject 7	Subject 8	Subject 9	Subject 10	Subject 11	Subject 12	Subject 13	Subject 14	Subject 15	Subject 16	Subject 17
**Sex**	M	M	M	M	F	M	M	M	M	M	M	M	M	M	M	M	M
**Current age**	26	34	23	25	23	26	26	22	23	14	21	21	21	15	13	14	12
**Nationality**	Qatar	Qatar	Qatar	Qatar	Qatar	Qatar	Qatar	Qatar	Qatar	Yemen	Qatar	Qatar	Qatar	Qatar	Qatar	Qatar	Palestine
**Developmental** **delay**	–	–	–	–	+	+	–	–	+	–	+	+	+	+	+	–	+
**Intellectual** **disability**	–	–	–	–	+	+	–	–	–	–	–	–	–	+	–	–	–
**Language/speech delay**	+	+	+	+	+	+	–	–	+	+	+	+	+	+	+	+	+
**Epilepsy/seizures/** **spasms**	–	+	–	–	–	–	–	+	+	–	+	–	–	–	–	–	–
**Brain MRI**	– normal	– normal	na	– normal	– normal	– normal	– normal	+	+	+	– normal	na	na	na	na	na	na
**EEG**	– normal	– normal	na	– normal	– normal	– normal	– normal	+ abnormal	+ abnormal	+ abnormal	+ abnormal	na	na	na	na	na	na
**Subject** **ID**	**Subject** **18**	**Subject 19**	**Subject 20**	**Subject 21**	**Subject** **22**	**Subject** **23**	**Subject** **24**	**Subject** **25**	**Subject** **26**	**Subject** **27**	**Subject** **28**	**Subject** **29**	**Subject** **30**	**Subject** **31**	**Subject** **32**	**Subject** **33**	**Subject** **34**
**Sex**	F	F	M	F	M	M	M	M	F	F	M	M	M	M	F	M	M
**Current age**	15	15	23	15	15	26	13	12	13	12	12	14	18	17	18	*15*	12
**Nationality**	Qatar	Qatar	Qatar	Tunisia	Qatar	Qatar	Qatar	Qatar	Qatar	Syria	Qatar	India	Qatar	Sudan	Jordan	Sudan	Syria
**Developmental** **delay**	–	–	–	–	+	–	–	–	–	+	–	–	–	–	–	–	–
**Intellectual** **disability**	–	–	+	+	–	–	+	+	–	+	–	+	+	–	–	–	–
**Language/speech delay**	+	–	+	+	+	–	+	–	–	+	–	+	+	–	–	+	–
**Epilepsy/seizures/** **spasms**	+	na	na	+	+	–	+	–	–	–	–	–	+	–	–	–	–
**Brain MRI**	– normal	na	– normal	– normal	+ abnormal	na	– normal	na	– normal	– normal	na	– normal	– normal	na	na	+ abnormal	na
**EEG**	+ abnormal	na	– normal	– normal	+ abnormal	– normal	NA	na	– normal	– normal	na	– normal	na	na	na	na	na
**Subject ID**	**Subject** **35**	**Subject 36**	**Subject 37**	**Subject 38**	**Subject** **39**	**Subject** **40**	**Subject** **41**	**Subject** **42**	**Subject** **43**	**Subject** **44**	**Subject** **45**		**Subject** **46**	**Subject** **47**	**Subject** **48**	**Subject** **49**	**Subject** **50**
**Sex**	M	F	M	M	M	M	M	M	M	M	M		M	F	F	M	M
**Current age**	14	13	12	19	11	16	13	12	15	13	13		13	11	22	22	12
**Nationality**	Syrian	Qatar	Algeria	Qatar	Egypt	Syrian	Qatar	Syrian	Qatar	Yemen	Qatar		Qatar	Qatar	Egypt	KSA	Qatar
**Developmental** **delay**	–	+	–	–	–	–	–	–	–	+	–		–	–	+	–	–
**Intellectual** **disability**	–	+	–	–	–	–	–	–	–	–	–		–	–	–	–	–
**Language/speech delay**	–	+	–	–	–	–	–	–	+	+	+		–	–	+	–	+`
**Epilepsy/seizures/** **spasms**	–	+	–	–	–	–	–	–	–	–	–		–	–	–	–	–
**Brain MRI**	– normal	– normal	na	– normal	– normal	– normal	– normal	na	– normal	– normal	– normal		na	– normal	na	– normal	– normal
**EEG**	– normal	+ abnormal	na	na	na	na	na	na	+ abnormal	na	na		na	na	na	– normal	– normal

The term ‘na’ is used to denote any unavailable data, while a dash (–) signifies the absence of the corresponding phenotype. EEG: electroencephalogram. Brain MRI: Magnetic Resonance Imaging. KSA: Kingdom of Saudi Arabia. Each column represents an individual with ASD, detailing their sex, age, and nationality, while each row lists accompanying phenotypes, including developmental delay, intellectual disability, language/speech delay, epilepsy/seizures/spasms, as well as brain MRI and EEG results.

**Table 2:** Overview of the GS analysis performed on single-nucleotide variants identified in 50 Qatari subjects diagnosed with ASD. A total of 70 genes were pinpointed through this GS analysis. Variants are denoted by their respective accession numbers, with ”NE” signifying the absence of information on a specific variant. Out of the 70 genes, 36 (marked in bold) are considered to have the highest likelihood of being causative for ASD based on criteria such as low prevalence, high-quality coverage, high CADD score, significant pLI and Z-scores, and alignment with ACMG guidelines. Notably, an expansion of more than 200 CGG repeats was found in the *FMR1* gene in subject 23. 13 Genes that are considered newly identified are emphasized in red.

Subject	Gender	Genes	Accession Number	Nucleotide Change	Protein Change	Type of Variant	Genotype	AF (GnomAD)	AF (QGP)	pLI Score	Z Score	CADD Score	ACMG Interpretation
1	Male	*SLC22A24*	NM_001136506.2	c.402+1G>A	splice	splice	homozygous	0	0.23686	0	−1.97	20.9	VUS
		*DMXL1*	NM_005509.6	c.4970+24delA	intronic	intronic	de novo	0	0	1	−0.16	NE	VUS
2	Male	***CUL2***	**NM_003591.4**	c.2057G>T	p.R686L	missense	de novo	0	0	1	2.55	29.9	LP
		*STXBP5L*	NM_014980.3	c.1673G>A	p.R558Q	missense	de novo	5.1 × 10^−5^	3.37 × 10^−3^	0.86	1.38	22.9	VUS
3	Male	***BAHD1***	**NM_014952.5**	c.1594G>A	p.A532T	missense	homozygous	0	3.06 × 10^−4^	1	1.82	25	LP
4	Male	*STK38*	NM_007271.4	c.589G>C	p.V197L	missense	de novo	0	0	0.02	2.36	23.1	VUS
		***DNAH3***	**NM_017539.2**	c.9565delG	p.V3189fs*7	frameshift	homozygous	0	0	0	2.47	NE	P
5	Female	*NEO1*	NM_002499.4	c.3688A>G	p.R1230G	missense	de novo	0	3.41 × 10^−5^	0.72	2.15	30	VUS
6	Male	***MAGEC1***	**NM_005462.5**	c.2415_2418dupTGCC	p.Q807fs*43	frameshift	X-linked	0	0	NE	NE	NE	P
7	Male	***CLIP1***	**NM_001389291.1**	c.4747T>G	p.L1583V	missense	homozygous	0	1.9 × 10^−3^	1	3.45	22	LP
		***PLXNB3***	**NM_005393.3**	c.5018G>A	p.R1673Q	missense	X-linked	1.7× 10^−5^	2.76 × 10^−3^	NE	NE	21.4	LP
8	Male	***ZNF746***	**NM_152557.5**	c.26T>C	p.I9T	missense	homozygous	0	1.8 × 10^−3^	0.86	1.13	25.1	LP
		***RNF133***	**NM_139175.2**	c.800A>G	p.D267G	missense	homozygous	2.4 × 10^−5^	3.54 × 10^−3^	0.35	0.09	25.5	LP
9	Male	*TNRC18*	NM_001080495.3	c.487+126delT	intronic	intronic	homozygous	0	1.5 × 10^−4^	0	−7.54	NE	LP
10	Male	***USP24***	**NM_015306.3**	c.70C>G	p.R24G	missense	de novo	0	0	1	4.12	28.2	LP
11	Male	*DET1*	NM_001144074.3	c.1416G>T	p.W472C	missense	de novo	0	0	0.01	0.4	25	VUS
12	Male	*GSTP1*	NM_000852.4	c.320T>A	p.L107H	missense	de novo	7.7 × 10^−5^	7.83 × 10^−4^	0	0.05	23.9	VUS
		*USH2A*	NM_206933.4	c.11389+14dupA	intronic	intronic	de novo	0	0	0	0.08	NE	VUS
13	Male	***RGS4***	**NM_001113381.1**	c.100G>C	p.E34Q	missense	homozygous	0	0	0	0.27	22.8	LP
		*PRR12*	NM_020719.3	c.2054G>C	p.G685A	missense	de novo	0	0	1	−1.05	23.3	VUS
14	Male	***FGF13***	**NM_033642.3**	c.545G>T	p.G182V	missense	X-linked	0	0	NE	NE	25	LP
15	Male	*KIF13B*	NM_015254.4	c.3256C>T	p.R1086*	nonsense	de novo	0	0	0	0.7	40	VUS
		*CADM3*	NM_021189.5	c.139G>A	p.E47K	missense	de novo	0	1.36 × 10^−4^	1	1.56	26.8	VUS
16	Male	*MANF*	NM_006010.6	c.59C>G	p.P20R	missense	de novo	0	2.15 × 10^−3^	0.03	−0.26	23.5	VUS
		***USF3***	**NM_001009899.4**	c.4416_4418delGCA	p.Q1478del	In-frame	homozygous	0	1.01 × 10^−3^	1	2.78	NE	LP
17	Male	***GRIA2***	**NM_001379001.3**	c.1740G>C	p.W580C	missense	de novo	0	0	1	5.48	26.2	LP
18	Female	***KCNK9***	**NM_001282534.2**	c.479T>C	p.V160A	missense	de novo	0	0	0.98	3.46	26.4	LP
		***NPTX1***	**NM_002522.4**	c.85C>T	p.R29C	missense	homozygous	0	2.39 × 10^−4^	0.96	1.6	32	LP
19	Female	*E2F8*	NM_024680.4	c.2480C>G	p.P827R	missense	de novo	0	0	0.42	1.54	26.5	VUS
20	Male	*AGBL4*	NM_032785.4	c.1195A>G	p.T399A	missense	de novo	0	0	0	0.58	27.6	VUS
21	Female	*DEAF1*	NM_021008.4	c.674G>T	p.G225V	missense	de novo	0	0	0	0.83	24.9	VUS
22	Male	***C12orf57***	**NM_001301834.1**	c.1A>G	p.M1V	start loss	homozygous	7.2 × 10^−5^	1.73 × 10^−3^	0	−1.26	22.4	P
23	Male	***FMR1***	**NM_002024.6**	>200 CGG repeats	>200 CGG repeats	CGG repeats	X-linked	NE	NE	NE	NE	NE	NE
24	Male	***SCRN2***	**NM_138355.4**	c.1061G>A	p.R354H	missense	homozygous	0	NE	0	0.66	26.2	LP
25	Male	*CYBC1*	NM_001193657.2	c.325G>C	p.D109H	missense	de novo	0	0	0	−0.13	23.4	VUS
		***KDM2A***	**NM_001256405.2**	c.1010A>G	p.K337R	missense	de novo	0	0	1	6.9	22.8	LP
26	Female	*VWA1*	NM_022834.5	c.1014C>G	p.I338M	missense	de novo	0	2.21 × 10^−3^	0	−3.94	22.1	VUS
		***LFNG***	**NM_001166355.2**	c.163_166dupGATG	p.E56fs*2	frameshift	homozygous	0	0	0.02	−0.15	NE	P
27	Female	*PTP4A1*	NM_001385266.1	c.439delT	p.S147fs*	frameshift	de novo	0	0	0.24	1.99	NE	VUS
		*TSPAN13*	NM_014399.4	c.61A>T	p.T21S	missense	de novo	0	0	0	1.28	24.3	VUS
28	Male	*ARL13B*	NM_182896.3	c.486+22dupT	intronic	intronic	de novo	0	0	0	0.13	NE	VUS
29	Male	*ATP8B3*	NM_138813.4	c.1552+182dupT	intronic	intronic	de novo	0	0	0	−1.72	NE	VUS
30	Male	*CACNA2D3*	NM_018398.3	c.1816T>G	p.Y606D	missense	de novo	0	0	1	1.54	27	VUS
31	Male	***DHX30***	**NM_014966.4**	c.1813-1G>T	splice	splice	de novo	0	0	1	6.9	34	LP
		***SLC12A8***	**NM_024628.6**	c.202A>G	p.N68D	missense	homozygous	0	0	0	0.8	26.2	LP
32	Female	*AKAP6*	NM_004274.5	c.347A>G	p.D116G	missense	de novo	0	0	1	1.11	28	VUS
		***RIN2***	**NM_001242581.2**	c.634A>G	p.I212V	missense	homozygous	0	0	0	0.88	25	LP
33	Male	***CHST7***	**NM_019886.4**	c.11G>A	p.R4Q	missense	X-linked	0	0	NE	NE	25.1	LP
34	Male	***TAF7L***	**NM_001168474.2**	c.247A>G	p.K83E	missense	X-linked	0	0	NE	NE	22.2	LP
35	Male	*CAPG*	NM_001747.4	c.668T>A	p.V223D	missense	de novo	0	0	0	0.62	31	VUS
36	Female	*KATNAL2*	NM_001353901.1	c.1273dupA	p.S425fs*11	frameshift	de novo	0	0	0	0.4	NE	VUS
37	Male	***FAAH2***	**NM_174912.4**	c.530G>A	p.S177N	missense	X-linked	0	0	NE	NE	22.7	LP
38	Male	***USP9X***	**NM_001039591.3**	c.6680A>G	p.K2227R	missense	X-linked	0	0	NE	NE	25.9	LP
39	Male	*C3orf38*	NM_173824.4	c.787G>T	p.E263*	nonsense	de novo	0	0	0	0.93	36	VUS
		*CTSZ*	NM_001336.4	c.700C>T	p.Q234*	nonsense	de novo	0	0	0	0.11	26.2	VUS
40	Male	*KLHL5*	NM_001171654.1	c.281G>T	p.G94V	missense	de novo	0	0	0	2.12	34	VUS
41	Male	***CCDC88C***	**NM_001080414.4**	c.4193C>T	p.P1398L	missense	homozygous	0	3.41 × 10^−5^	0	−0.41	24.5	LP
		***AP5Z1***	**NM_014855.3**	c.70T>C	p.F24L	missense	homozygous	1.3 × 10^−5^	4.77 × 10^−4^	0	−6.12	21.8	LP
42	Male	***OBSL1***	**NM_001173408.2**	c.2954A>T	p.E985V	missense	homozygous	0	0	0	−1.07	22.4	LP
43	Male	*FOXK1*	NM_001037165.2	c.674T>G	p.L225R	missense	de novo	0	0	0.04	0.15	29.3	VUS
44	Male	***CNKSR2***	**NM_001330772.2**	c.2242G>C	p.A748P	missense	X-linked	0	0	NE	NE	20.2	LP
45	Male	*ZSCAN25*	NM_145115.3	c.848A>C	p.D283A	missense	de novo	0	0	0	1.04	20.6	VUS
		***FRMPD3***	**NM_032428.2**	c.2870A>G	p.E957G	missense	X-linked	0	1.63 × 10^−3^	NE	NE	25.6	LP
46	Male	***ARSF***	**NM_004042.5**	c.1408G>T	p.A470S	missense	X-linked	0	2.04 × 10^−4^	NE	NE	22.8	LP
		*POLQ*	NM_199420.4	c.5774-5dupT	intronic	intronic	de novo	0	0	0	1.62	NE	VUS
47	Female	***SLC25A42***	**NM_178526.5**	c.383C>G	p.A128G	missense	homozygous	0	1.36 × 10^−4^	0	1.24	20.2	LP
		*FTSJ1*	NM_177439.3	c.131G>A	p.R44Q	missense	X-linked	0	1.02 × 10^−4^	NE	NE	26.4	VUS
48	Female	***CSMD1***	**NM_033225.6**	c.5432G>A	p.G1811D	missense	comp. het	0	3.41 × 10^−5^	0	−12.45	23.5	LP
				c.5185G>C	p.V1729L	missense		1.93 × 10^−4^	3.41 × 10^−5^			21.6	LP
49	Male	***KCNC4***	**NM_004978.6**	c.712G>T	p.V238F	missense	homozygous	0	6.82 × 10^−5^	0	2.02	22.8	LP
50	Male	***GRIN3B***	**NM_138690.3**	c.1571G>A	p.R524Q	missense	homozygous	0	1.84 × 10^−3^	0	−5.07	21.5	LP

Variants are classified as VUS (variant of unknown significance), LP (likely pathogenic), and P (pathogenic). AF (allele frequency): the frequency of the variant in the general population, indicating how common or rare the variant is in (gnomAD) and the Qatar Genome Project (QGP). pLI Score (probability of being loss-of-function intolerant): a score that estimates the likelihood that a gene can tolerate disruptions without leading to severe phenotypes; a higher score suggests greater intolerance to loss-of-function mutations, indicating the gene’s potential involvement in disease. The Z-score measures the deviation of the observed number of missense variants in a gene from the expected number, indicating the gene’s tolerance or intolerance to such variations. The CADD score (Combined Annotation Dependent Depletion score) predicts the potential deleteriousness of genetic variants, with higher scores indicating a greater likelihood that the variant adversely affects gene function and contributes to disease. The ACMG interpretation refers to guidelines established by the American College of Medical Genetics and Genomics for classifying genetic variants into categories such as benign, likely benign, uncertain significance, likely pathogenic, and pathogenic, aiding in the assessment of their clinical significance in relation to diseases.

## Data Availability

The data is not publicly available as the data is protected and inaccessible due to privacy regulations that aim to safeguard the privacy and consent of research participants. However, the data that supports the findings of this study is available upon request from the corresponding authors.

## Supplementary Materials

The following supporting information can be downloaded at https://www.mdpi.com/article/10.3390/ijms252111551/s1.

## Abbreviations

autism spectrum disorder (ASD), neurodevelopmental disorders (NDDs), development delay (DD), intellectual disability (ID), next-generation sequencing (NGS), Middle East and North Africa (MENA), genome analysis toolkit (GATK), American College of Medical Genetics and Genomics/Association for Molecular Pathology (ACMG/AMP), human gene mutation database (HGMD), exome sequencing (ES), genome sequencing (GS), single nucleotide variants (SNVs), single-nucleotide polymorphism (SNP), copy number variants (CNVs), structural variants (SVs), tandem repeats expansions (TREs), variant of uncertain significance (VUS), Qatar Genome Project (QGP), Qatar Biobank (QBB), minor allele frequencies (MAFs).
