# Supplementary material for: Genome Sequencing Identifies 13 Novel Candidate Risk Genes for Autism Spectrum Disorder in a Qatari Cohort
Source: Int J Mol Sci. 2024 Oct 27;25(21):11551. doi: 10.3390/ijms252111551 (PMC11547081; doi:10.3390/ijms252111551)
Supplement: Supplementary file 1 [file ijms-25-11551-s001.zip › ijms-3275327-supplementary.pdf]

Subject ID	Gene	Gene description	Gene in HGMD for reported pathogenic variants	Gene in SFARI	Gene in NDD
2	***CUL2***	Cullin 2	1. Autism [1, 2]<br>2. Developmental disorder [3]	NP	NP
3	***BAHD1***	Bromo adjacent homology domain containing 1	1. Severe developmental delay, neurological features and dysmorphism [4]<br>2. Autism spectrum disorder [1, 5]<br>3. Autism [1]<br>4. Developmental disorder [1, 3]	NP	NP
4	*DNAH3*	Dynein axonemal heavy chain 3	1. Developmental disorder [1, 3, 6]<br>2. Autism spectrum disorder [1, 5]<br>3. Autism [6]<br>4. Autism spectrum disorder [7]<br>5. Intellectual disability [8, 9]	P	NP
6	*MAGEC1*	MAGE family member C1	1. Developmental disorder [10]<br>2. Autism [1, 6]<br>3. Autism spectrum disorder [6, 11]<br>4. Schizophrenia [12]<br>5. Developmental disorder [1, 3]	NP	P
7	*PLXNB3*	Plexin B3	1. Developmental disorder [6, 10]<br>2. Autism spectrum disorder [6, 11]<br>3. Autism [1]<br>4. Hydrocephalus [13]<br>5. Intellectual disability [14]	NP	P
	CLIP1	*CAP-GLY domain containing linker protein 1*	1. Developmental and epileptic encephalopathy [15]<br>2. Developmental disorder [6]<br>3. Autism spectrum disorder [16]<br>4. Autism [1]<br>5. Intellectual disability [17]	NP	NP
8	***ZNF746***	zinc finger protein 746	1. Early-onset parkinson disease, association with [18]<br>2. Developmental disorder [1, 3]	NP	NP
	RNF133	ring finger protein 133	1. Autism spectrum disorder [5]<br>2. Developmental disorder [1, 3]	NP	NP
10	***USP24***	Ubiquitin specific peptidase 24	1. Parkinson disease [19]<br>2. Developmental disorder [1, 3]<br>3. Autism spectrum disorder [1, 5, 11, 16, 20, 21]<br>4. Attention-deficit hyperactivity disorder [22]<br>5. Epilepsy [23]<br>6. Developmental disorder [6]	NP	NP
13	***RGS4***	Regulator of G protein signaling 4	1. Developmental disorder [1, 3]	NP	NP
14	*FGF13*	Fibroblast growth factor 13	1. Developmental and epileptic encephalopathy [10, 24-26]<br>2. Epilepsy with febrile seizures plus [27]	P	P
16	*USF3*	Upstream transcription factor family member 3	1. Autism spectrum disorder [1, 5, 16, 20]<br>2. Childhood disintegrative disorder [28, 29]<br>3. Cleft palate, dysmorphic features and mild developmental delay, 3 healthy daughters, twins stillbirth at 22 m [30]<br>4. Schizophrenia [31]	NP	NP
17	*GRIA2*	Glutamate ionotropic receptor AMPA type subunit 2	1. Intellectual disability, autism spectrum disorder, seizures and speech impairment [32]<br>2. Schizophrenia [33]<br>3. Intellectual disability [32, 34]<br>4. Intellectual disability, seizures and speech impairment [32]<br>5. Autism [6, 35]<br>6. Autism spectrum disorder [1, 5, 16, 20, 36]<br>7. Cognitive impairment [37]<br>8. Neurodevelopmental disorder with language impairment [38]<br>9. Seizures [32]	P	P
18	*NPTX1*	Neuronal pentraxin 1	1. Cerebellar ataxia [39-42]<br>2. Developmental disorder [1, 3]	NP	NP
	KCNK9	Potassium two pore domain channel subfamily K member 9	1. Birk Barel mental retardation dysmorphism syndrome [43-47]<br>2. KCNK9 imprinting syndrome [48]<br>3. Autism spectrum disorder [49]<br>4. Cerebral palsy [50]	NP	P
22	*C12orf57*	Chromosome 12 open reading frame 57	1. Epilepsy [51]<br>2. Autism spectrum disorder [1, 5, 52]	P	P

			3. Temtamy syndrome [53-55]<br>4. Intellectual disability [56-58]<br>5. Developmental delay, intellectual disability, ADHD and bladder/kidney abnormalities [55, 59, 60]<br>6. Severe global developmental delay [55, 60, 61]<br>7. Intellectual disability, hypoplasia of corpus callosum, chorioretinal coloboma and intractable seizures [62]		
23	*FMR1*	Fragile X mental retardation 1	1. Fragile X mental retardation syndrome [63-67]<br>2. Intellectual disability [68-71]<br>3. Neurodevelopmental dysfunction [72]<br>4. Intellectual disability, developmental delay, macrocephaly and speech delay [73]<br>5. Neurodevelopmental disorder [74]<br>6. Autism spectrum disorder [75, 76]<br>7. Developmental delay, epilepsy and hyperactivity [77]<br>8. Intellectual disability and seizure disorder [78]<br>9. Intellectual disability, developmental delay and epilepsy [79]<br>10. Parkinson disease [80]<br>11. Seizure [81]	P	P
24	***SCRN2***	secernin 2	1. Autism spectrum disorder [1, 5, 6, 20]<br>2. Developmental disorder [1, 3]	NP	NP
25	*KDM2A*	Lysine demethylase 2A	1. Autism spectrum disorder [1, 5, 6, 11, 16, 20, 82]<br>2. Schizophrenia [83]<br>3. Developmental disorder [1, 3]	P	NP
26	***LFNG***	LFNG O-fucosylpeptide 3-beta-N-acetylglucosaminyltransferase	1. Asperger syndrome [84]<br>2. Autism spectrum disorder [7]<br>3. Autism [1]	NP	P
31	*DHX30*	DExH-box helicase 30	1. Neurodevelopmental disorder [85-88]<br>2. Autism spectrum disorder [1, 5]<br>3. Global developmental delay, intellectual disability, speech impairment and gait abnormalities [16, 89, 90]<br>4. Developmental delay/intellectual disability, hypotonia and microcephaly [89, 91]<br>5. Seizures, microcephaly and short stature [89, 91]	P	P
	SLC12A8	solute carrier family 12 member 8	1. Autism spectrum disorder [1, 5]	NP	NP
32	*RIN2*	Ras and Rab interactor 2	1. Macrocephaly, alopecia, cutis laxa and scoliosis [92, 93]<br>2. RIN2 syndrome [94-96]<br>3. Autism spectrum disorder [5, 6, 11, 20]<br>4. Macrocephaly, motor and speech delay, intellectual disability, learning disability, periventricular leukomalacia, epilepsy [30]<br>5. Autism [1]	NP	NP
33	***CHST7***	carbohydrate sulfotransferase 7	1. Autism [1]<br>2. Developmental disorder [1, 3]	NP	NP
34	*TAF7L*	TATA-box binding protein associated factor 7 like	1. Developmental disorder [1, 3, 10]<br>2. Schizophrenia [97]<br>3. West syndrome [98]	NP	P
37	*FAAH2*	Fatty acid amide hydrolase 2	1. Autism spectrum disorder [6, 11, 99]<br>2. Developmental disorder [6]<br>3. Intellectual disability, dysmorphic features [30]<br>4. Intellectual disability, X-linked [100]<br>5. Neurologic and psychiatric disorders [101, 102]<br>6. Seizures, learning disability [30]<br>7. Zellweger syndrome [103]	NP	P
38	*USP9X*	Ubiquitin specific peptidase 9 X-linked	1. Neurodevelopmental disorder [54, 104, 105]<br>2. Developmental delay and congenital malformations [6, 16, 90]<br>3. Intellectual disability [106]<br>4. Autism spectrum disorder [6, 11, 16, 54, 107]<br>5. Epileptic encephalopathy [108]<br>6. Mental retardation [102, 109]<br>7. Ataxia [110]<br>8. Autism [1, 6]<br>9. Developmental disorder [10]<br>10. Failure to thrive, growth retardation, microcephaly, motor and speech delay, intellectual disability, nystagmus, spasticity [30]<br>11. Epilepsy [111]	P	P

41	*AP5Z1*	Adaptor related protein complex 5 subunit zeta 1	1. Schizophrenia [112]<br>2. Ataxia [113]<br>3. Autism [6]<br>4. Autism spectrum disorder [1, 5]<br>5. Fine/gross motor delay, Speech delay, Intellectual disability, Learning disability, Developmental regression, Autism Spectrum Disorders, Seizures [30]<br>6. Spastic quadriplegia, diminished cerebral volumes with delayed myelination, global developmental delay, microcephaly and epilepsy [114]	NP	P
	CCDC88C	Coiled-coil domain containing 88C	1. Hydrocephalus [115-118]<br>2. Spinocerebellar ataxia 40 [119-124]<br>3. Spastic ataxia [125]<br>4. Autism spectrum disorder [11, 20, 36, 126, 127]<br>5. Spinocerebellar ataxia [124, 128]<br>6. Autism [129]<br>7. Developmental disorder [6]<br>8. Hydrocephalus [117]<br>9. Spastic paraplegia, early-onset progressive [130]	P	P
42	*OBSL1*	Obscurin like 1	1. 3-M syndrome [131]<br>2. Autism spectrum disorder [1, 5]<br>3. Dwarfism [132];<br>4. Short stature [133]<br>5. Autism [6, 134]<br>6. Bipolar disorder [135]<br>7. Developmental disorder [6]<br>8. Obsessive-compulsive disorder [136]	NP	P
44	*CNKSR2*	Connector enhancer of kinase suppressor of Ras 2	1. Neurodevelopmental and epilepsy disorder [137]<br>2. Encephalopathy with status epilepticus during slow sleep [138, 139]<br>3. Developmental disorder [6, 90]<br>4. Intellectual disability, X-linked non-syndromic [140]<br>5. Neurodevelopmental disorder [54]<br>6. Autism spectrum disorder [141]<br>7. Epilepsy, childhood-onset [142]<br>8. Intellectual disability, X-linked, syndromic [143]<br>9. Psychomotor delay and ADHD [144]<br>10. Seizures and intellectual disability [145]	P	P
45	***FRMPD3***	FERM and PDZ domain containing 3	1. West syndrome [98]<br>2. Autism [1]<br>3. Developmental disorder [3]	NP	NP
46	***ARSF***	Arylsulfatase F	1. Autism spectrum disorder [99]<br>2. West syndrome [98]<br>3. Developmental disorder [1, 3]	NP	P
47	*SLC25A42*	Solute carrier family 25 member 42	1. Encephalopathy, epileptic [146]<br>2. Autism spectrum disorder [1, 5]<br>3. Mitochondrial myopathy [146-150]	NP	NP
48	*CSMD1*	CUB and Sushi multiple domains 1	1. Autism spectrum disorder [36, 126] [49]<br>2. Schizophrenia [151-154]<br>3. Developmental disorder [6]<br>4. Autism/schizophrenia [155]<br>5. Parkinson disease [156]<br>6. Attention deficit hyperactivity disorder [157]<br>7. Autism [158]<br>8. Developmental delay [159]<br>9. Developmental delay and autism spectrum disorder/autistic features[159]<br>10. Intellectual disability, developmental delay, autism and ADHD [160]<br>11. Intellectual disability and autism spectrum disorder [161]<br>12. Schizophrenia [162]<br>13. Speech delay [159]<br>14. Speech delay and autistic features [160]<br>15. Speech delay and autism spectrum disorder/autistic features [159]	P	NP

49	*KCNC4*	Potassium voltage-gated channel subfamily C member 4	1. Ataxia and dystonia [60]<br>2. Autism spectrum disorder [1, 5]<br>3. Bipolar disorder [135]<br>4. Developmental disorder [1, 3, 6, 90]	NP	NP
50	*GRIN3B*	Glutamate ionotropic receptor NMDA type subunit 3B	1. Schizophrenia [163-165]<br>2. Autism [1, 5, 6]<br>3. Autism spectrum disorder [163]<br>4. Schizophrenia and autism spectrum disorder [163]<br>5. Developmental disorder [1, 3]	NP	NP

Table S1. List of the 37 candidate genes identified as most likely causative for ASD in our study. These genes, validated through extensive literature review and databases like the Human Gene Mutation Database (HGMD), were also cross-referenced with international autism and NDD gene panel from Genomics England NDD/autism panel genes *(* https://panelapp.genomicsengland.co.uk/panels/285/) and Simons Foundation Autism Research Initiative (SFARI) (https://gene.sfari.org/database/human-gene/). The presence of a gene in these lists is denoted by 'P' and absence by 'NP'. 13 Genes considered newly identified are emphasized in red.

REFERENCES

1. Zhou, X.; Feliciano, P.; Shu, C.; Wang, T.; Astrovskaya, I.; Hall, J. B.; Obiajulu, J. U.; Wright, J. R.; Murali, S. C.; Xu, S. X.; Brueggeman, L.; Thomas, T. R.; Marchenko, O.; Fleisch, C.; Barns, S. D.; Snyder, L. G.; Han, B.; Chang, T. S.; Turner, T. N.; Harvey, W. T.; Nishida, A.; O'Roak, B. J.; Geschwind, D. H.; Consortium, S.; Michaelson, J. J.; Volfovsky, N.; Eichler, E. E.; Shen, Y.; Chung, W. K., Integrating de novo and inherited variants in 42,607 autism cases identifies mutations in new moderate-risk genes. *Nat Genet* **2022,** 54, (9), 1305-1319.
2. Wilfert, A. B.; Turner, T. N.; Murali, S. C.; Hsieh, P.; Sulovari, A.; Wang, T.; Coe, B. P.; Guo, H.; Hoekzema, K.; Bakken, T. E.; Winterkorn, L. H.; Evani, U. S.; Byrska-Bishop, M.; Earl, R. K.; Bernier, R. A.; Consortium, S.; Zody, M. C.; Eichler, E. E., Recent ultra-rare inherited variants implicate new autism candidate risk genes. *Nat Genet* **2021,** 53, (8), 1125-1134.
3. Kaplanis, J.; Samocha, K. E.; Wiel, L.; Zhang, Z.; Arvai, K. J.; Eberhardt, R. Y.; Gallone, G.; Lelieveld, S. H.; Martin, H. C.; McRae, J. F.; Short, P. J.; Torene, R. I.; de Boer, E.; Danecek, P.; Gardner, E. J.; Huang, N.; Lord, J.; Martincorena, I.; Pfundt, R.; Reijnders, M. R. F.; Yeung, A.; Yntema, H. G.; Deciphering Developmental Disorders, S.; Vissers, L.; Juusola, J.; Wright, C. F.; Brunner, H. G.; Firth, H. V.; FitzPatrick, D. R.; Barrett, J. C.; Hurles, M. E.; Gilissen, C.; Retterer, K., Evidence for 28 genetic disorders discovered by combining healthcare and research data. *Nature* **2020,** 586, (7831), 757-762.
4. Murcia Pienkowski, V.; Kucharczyk, M.; Mlynek, M.; Szczaluba, K.; Rydzanicz, M.; Poszewiecka, B.; Skorka, A.; Sykulski, M.; Biernacka, A.; Koppolu, A. A.; Posmyk, R.; Walczak, A.; Kosinska, J.; Krajewski, P.; Castaneda, J.; Obersztyn, E.; Jurkiewicz, E.; Smigiel, R.; Gambin, A.; Chrzanowska, K.; Krajewska-Walasek, M.; Ploski, R., Mapping of breakpoints in balanced chromosomal translocations by shallow whole-genome sequencing points to EFNA5, BAHD1 and PPP2R5E as novel candidates for genes causing human Mendelian disorders. *J Med Genet* **2019,** 56, (2), 104-112.
5. Fu, J. M.; Satterstrom, F. K.; Peng, M.; Brand, H.; Collins, R. L.; Dong, S.; Wamsley, B.; Klei, L.; Wang, L.; Hao, S. P.; Stevens, C. R.; Cusick, C.; Babadi, M.; Banks, E.; Collins, B.; Dodge, S.; Gabriel, S. B.; Gauthier, L.; Lee, S. K.; Liang, L.; Ljungdahl, A.; Mahjani, B.; Sloofman, L.; Smirnov, A. N.; Barbosa, M.; Betancur, C.; Brusco, A.; Chung, B. H. Y.; Cook, E. H.; Cuccaro, M. L.; Domenici, E.; Ferrero, G. B.; Gargus, J. J.; Herman, G. E.; Hertz-Picciotto, I.; Maciel, P.; Manoach, D. S.; Passos-Bueno, M. R.; Persico, A. M.; Renieri, A.; Sutcliffe, J. S.; Tassone, F.; Trabetti, E.; Campos, G.; Cardaropoli, S.; Carli, D.; Chan, M. C. Y.; Fallerini, C.; Giorgio, E.; Girardi, A. C.; Hansen-Kiss, E.; Lee, S. L.; Lintas, C.; Ludena, Y.; Nguyen, R.; Pavinato, L.; Pericak-Vance, M.; Pessah, I. N.; Schmidt, R. J.; Smith, M.; Costa, C. I. S.; Trajkova, S.; Wang, J. Y. T.; Yu, M. H. C.; Autism Sequencing, C.; Broad Institute Center for Common Disease, G.; i, P.-B. C.; Cutler, D. J.; De Rubeis, S.; Buxbaum, J. D.; Daly, M. J.; Devlin, B.; Roeder, K.; Sanders, S. J.; Talkowski, M. E., Rare coding variation provides insight into the genetic architecture and phenotypic context of autism. *Nat Genet* **2022,** 54, (9), 1320-1331.
6. Turner, T. N.; Wilfert, A. B.; Bakken, T. E.; Bernier, R. A.; Pepper, M. R.; Zhang, Z.; Torene, R. I.; Retterer, K.; Eichler, E. E., Sex-Based Analysis of De Novo Variants in Neurodevelopmental Disorders. *Am J Hum Genet* **2019,** 105, (6), 1274-1285.
7. Wu, J.; Yu, P.; Jin, X.; Xu, X.; Li, J.; Li, Z.; Wang, M.; Wang, T.; Wu, X.; Jiang, Y.; Cai, W.; Mei, J.; Min, Q.; Xu, Q.; Zhou, B.; Guo, H.; Wang, P.; Zhou, W.; Hu, Z.; Li, Y.; Cai, T.; Wang, Y.; Xia, K.; Jiang, Y. H.; Sun, Z. S., Genomic landscapes of Chinese sporadic autism spectrum disorders revealed by whole-genome sequencing. *J Genet Genomics* **2018,** 45, (10), 527-538.
8. Kahrizi, K.; Hu, H.; Hosseini, M.; Kalscheuer, V. M.; Fattahi, Z.; Beheshtian, M.; Suckow, V.; Mohseni, M.; Lipkowitz, B.; Mehvari, S.; Mehrjoo, Z.; Akhtarkhavari, T.; Ghaderi, Z.; Rahimi, M.; Arzhangi, S.; Jamali, P.; Falahat Chian, M.; Nikuei, P.; Sabbagh Kermani, F.; Sadeghinia, F.; Jazayeri, R.; Tonekaboni, S. H.; Khoshaeen, A.; Habibi, H.; Pourfatemi, F.; Mojahedi, F.; Khodaie-Ardakani, M. R.; Najafipour, R.; Wienker, T. F.; Najmabadi, H.; Ropers, H. H., Effect of inbreeding on intellectual disability revisited by trio sequencing. *Clin Genet* **2019,** 95, (1), 151-159.
9. Jarvela, I.; Maatta, T.; Acharya, A.; Leppala, J.; Jhangiani, S. N.; Arvio, M.; Siren, A.; Kankuri-Tammilehto, M.; Kokkonen, H.; Palomaki, M.; Varilo, T.; Fang, M.; Hadley, T. D.; Jolly, A.; Linnankivi, T.; Paetau, R.; Saarela, A.; Kalviainen, R.; Olme, J.; Nouel-Saied, L. M.; Cornejo-Sanchez, D. M.; Llaci, L.; Lupski, J. R.; Posey, J. E.; Leal, S. M.; Schrauwen, I., Exome sequencing reveals predominantly de novo variants in

disorders with intellectual disability (ID) in the founder population of Finland. *Hum Genet* **2021,** 140, (7), 1011-1029.

10. Martin, H. C.; Gardner, E. J.; Samocha, K. E.; Kaplanis, J.; Akawi, N.; Sifrim, A.; Eberhardt, R. Y.; Tavares, A. L. T.; Neville, M. D. C.; Niemi, M. E. K.; Gallone, G.; McRae, J.; Deciphering Developmental Disorders, S.; Wright, C. F.; FitzPatrick, D. R.; Firth, H. V.; Hurles, M. E., The contribution of X-linked coding variation to severe developmental disorders. *Nat Commun* **2021,** 12, (1), 627.
11. Iossifov, I.; O'Roak, B. J.; Sanders, S. J.; Ronemus, M.; Krumm, N.; Levy, D.; Stessman, H. A.; Witherspoon, K. T.; Vives, L.; Patterson, K. E.; Smith, J. D.; Paeper, B.; Nickerson, D. A.; Dea, J.; Dong, S.; Gonzalez, L. E.; Mandell, J. D.; Mane, S. M.; Murtha, M. T.; Sullivan, C. A.; Walker, M. F.; Waqar, Z.; Wei, L.; Willsey, A. J.; Yamrom, B.; Lee, Y. H.; Grabowska, E.; Dalkic, E.; Wang, Z.; Marks, S.; Andrews, P.; Leotta, A.; Kendall, J.; Hakker, I.; Rosenbaum, J.; Ma, B.; Rodgers, L.; Troge, J.; Narzisi, G.; Yoon, S.; Schatz, M. C.; Ye, K.; McCombie, W. R.; Shendure, J.; Eichler, E. E.; State, M. W.; Wigler, M., The contribution of de novo coding mutations to autism spectrum disorder. *Nature* **2014,** 515, (7526), 216-21.
12. Xu, B.; Roos, J. L.; Dexheimer, P.; Boone, B.; Plummer, B.; Levy, S.; Gogos, J. A.; Karayiorgou, M., Exome sequencing supports a de novo mutational paradigm for schizophrenia. *Nat Genet* **2011,** 43, (9), 864-8.
13. Jin, S. C.; Dong, W.; Kundishora, A. J.; Panchagnula, S.; Moreno-De-Luca, A.; Furey, C. G.; Allocco, A. A.; Walker, R. L.; Nelson-Williams, C.; Smith, H.; Dunbar, A.; Conine, S.; Lu, Q.; Zeng, X.; Sierant, M. C.; Knight, J. R.; Sullivan, W.; Duy, P. Q.; DeSpenza, T.; Reeves, B. C.; Karimy, J. K.; Marlier, A.; Castaldi, C.; Tikhonova, I. R.; Li, B.; Pena, H. P.; Broach, J. R.; Kabachelor, E. M.; Ssenyonga, P.; Hehnly, C.; Ge, L.; Keren, B.; Timberlake, A. T.; Goto, J.; Mangano, F. T.; Johnston, J. M.; Butler, W. E.; Warf, B. C.; Smith, E. R.; Schiff, S. J.; Limbrick, D. D., Jr.; Heuer, G.; Jackson, E. M.; Iskandar, B. J.; Mane, S.; Haider, S.; Guclu, B.; Bayri, Y.; Sahin, Y.; Duncan, C. C.; Apuzzo, M. L. J.; DiLuna, M. L.; Hoffman, E. J.; Sestan, N.; Ment, L. R.; Alper, S. L.; Bilguvar, K.; Geschwind, D. H.; Gunel, M.; Lifton, R. P.; Kahle, K. T., Exome sequencing implicates genetic disruption of prenatal neuro-gliogenesis in sporadic congenital hydrocephalus. *Nat Med* **2020,** 26, (11), 1754-1765.
14. Athanasakis, E.; Licastro, D.; Faletra, F.; Fabretto, A.; Dipresa, S.; Vozzi, D.; Morgan, A.; d'Adamo, A. P.; Pecile, V.; Biarnes, X.; Gasparini, P., Next generation sequencing in nonsyndromic intellectual disability: from a negative molecular karyotype to a possible causative mutation detection. *Am J Med Genet A* **2014,** 164A, (1), 170-6.
15. Takata, A.; Nakashima, M.; Saitsu, H.; Mizuguchi, T.; Mitsuhashi, S.; Takahashi, Y.; Okamoto, N.; Osaka, H.; Nakamura, K.; Tohyama, J.; Haginoya, K.; Takeshita, S.; Kuki, I.; Okanishi, T.; Goto, T.; Sasaki, M.; Sakai, Y.; Miyake, N.; Miyatake, S.; Tsuchida, N.; Iwama, K.; Minase, G.; Sekiguchi, F.; Fujita, A.; Imagawa, E.; Koshimizu, E.; Uchiyama, Y.; Hamanaka, K.; Ohba, C.; Itai, T.; Aoi, H.; Saida, K.; Sakaguchi, T.; Den, K.; Takahashi, R.; Ikeda, H.; Yamaguchi, T.; Tsukamoto, K.; Yoshitomi, S.; Oboshi, T.; Imai, K.; Kimizu, T.; Kobayashi, Y.; Kubota, M.; Kashii, H.; Baba, S.; Iai, M.; Kira, R.; Hara, M.; Ohta, M.; Miyata, Y.; Miyata, R.; Takanashi, J. I.; Matsui, J.; Yokochi, K.; Shimono, M.; Amamoto, M.; Takayama, R.; Hirabayashi, S.; Aiba, K.; Matsumoto, H.; Nabatame, S.; Shiihara, T.; Kato, M.; Matsumoto, N., Comprehensive analysis of coding variants highlights genetic complexity in developmental and epileptic encephalopathy. *Nat Commun* **2019,** 10, (1), 2506.
16. Kosmicki, J. A.; Samocha, K. E.; Howrigan, D. P.; Sanders, S. J.; Slowikowski, K.; Lek, M.; Karczewski, K. J.; Cutler, D. J.; Devlin, B.; Roeder, K.; Buxbaum, J. D.; Neale, B. M.; MacArthur, D. G.; Wall, D. P.; Robinson, E. B.; Daly, M. J., Refining the role of de novo protein-truncating variants in neurodevelopmental disorders by using population reference samples. *Nat Genet* **2017,** 49, (4), 504-510.
17. Larti, F.; Kahrizi, K.; Musante, L.; Hu, H.; Papari, E.; Fattahi, Z.; Bazazzadegan, N.; Liu, Z.; Banan, M.; Garshasbi, M.; Wienker, T. F.; Hilger Ropers, H.; Galjart, N.; Najmabadi, H., A defect in the CLIP1 gene (CLIP-170) can cause autosomal recessive intellectual disability. *Eur J Hum Genet* **2015,** 23, (3), 416.
18. Li, C. Y.; Ou, R. W.; Chen, Y. P.; Gu, X. J.; Wei, Q. Q.; Cao, B.; Zhang, L. Y.; Hou, Y. B.; Liu, K. C.; Chen, X. P.; Song, W.; Zhao, B.; Wu, Y.; Liu, Y.; Shang, H. F., Genetic Analysis of ZNF Protein Family Members for Early-Onset Parkinson's Disease in Chinese Population. *Mol Neurobiol* **2021,** 58, (7), 3435-3442.
19. Li, Y.; Schrodi, S.; Rowland, C.; Tacey, K.; Catanese, J.; Grupe, A., Genetic evidence for ubiquitin-specific proteases USP24 and USP40 as candidate genes for late-onset Parkinson disease. *Hum Mutat* **2006,** 27, (10), 1017-23.

20. Lim, E. T.; Uddin, M.; De Rubeis, S.; Chan, Y.; Kamumbu, A. S.; Zhang, X.; D'Gama, A. M.; Kim, S. N.; Hill, R. S.; Goldberg, A. P.; Poultney, C.; Minshew, N. J.; Kushima, I.; Aleksic, B.; Ozaki, N.; Parellada, M.; Arango, C.; Penzol, M. J.; Carracedo, A.; Kolevzon, A.; Hultman, C. M.; Weiss, L. A.; Fromer, M.; Chiocchetti, A. G.; Freitag, C. M.; Autism Sequencing, C.; Church, G. M.; Scherer, S. W.; Buxbaum, J. D.; Walsh, C. A., Rates, distribution and implications of postzygotic mosaic mutations in autism spectrum disorder. *Nat Neurosci* **2017,** 20, (9), 1217-1224.
21. Koire, A.; Katsonis, P.; Kim, Y. W.; Buchovecky, C.; Wilson, S. J.; Lichtarge, O., A method to delineate de novo missense variants across pathways prioritizes genes linked to autism. *Sci Transl Med* **2021,** 13, (594).
22. Liu, Y.; Chang, X.; Qu, H. Q.; Tian, L.; Glessner, J.; Qu, J.; Li, D.; Qiu, H.; Sleiman, P.; Hakonarson, H., Rare Recurrent Variants in Noncoding Regions Impact Attention-Deficit Hyperactivity Disorder (ADHD) Gene Networks in Children of both African American and European American Ancestry. *Genes (Basel)* **2021,** 12, (2).
23. Jabbari, K.; Bobbili, D. R.; Lal, D.; Reinthaler, E. M.; Schubert, J.; Wolking, S.; Sinha, V.; Motameny, S.; Thiele, H.; Kawalia, A.; Altmuller, J.; Toliat, M. R.; Kraaij, R.; van Rooij, J.; Uitterlinden, A. G.; Ikram, M. A.; Euro, E. C. C.; Zara, F.; Lehesjoki, A. E.; Krause, R.; Zimprich, F.; Sander, T.; Neubauer, B. A.; May, P.; Lerche, H.; Nurnberg, P., Rare gene deletions in genetic generalized and Rolandic epilepsies. *PLoS One* **2018,** 13, (8), e0202022.
24. Fry, A. E.; Marra, C.; Derrick, A. V.; Pickrell, W. O.; Higgins, A. T.; Te Water Naude, J.; McClatchey, M. A.; Davies, S. J.; Metcalfe, K. A.; Tan, H. J.; Mohanraj, R.; Avula, S.; Williams, D.; Brady, L. I.; Mesterman, R.; Tarnopolsky, M. A.; Zhang, Y.; Yang, Y.; Wang, X.; Genomics England Research, C.; Rees, M. I.; Goldfarb, M.; Chung, S. K., Missense variants in the N-terminal domain of the A isoform of FHF2/FGF13 cause an X-linked developmental and epileptic encephalopathy. *Am J Hum Genet* **2021,** 108, (1), 176-185.
25. Narayanan, D. L.; Majethia, P.; Shrikiran, A.; Siddiqui, S.; Dalal, A.; Shukla, A., Further evidence of affected females with a heterozygous variant in FGF13 causing X-linked developmental and epileptic encephalopathy 90. *Eur J Med Genet* **2022,** 65, (1), 104403.
26. Chen, J.; Chen, Y.; Yang, Y.; Niu, X.; Zhang, J.; Zeng, Q.; Liu, A.; Xu, X.; Yang, X.; Li, S.; Yang, X.; Wang, Y.; Zhang, Y., Detecting genomic mosaicism in "de novo" genetic epilepsy by amplicon-based deep sequencing. *J Hum Genet* **2023,** 68, (2), 73-80.
27. Puranam, R. S.; He, X. P.; Yao, L.; Le, T.; Jang, W.; Rehder, C. W.; Lewis, D. V.; McNamara, J. O., Disruption of Fgf13 causes synaptic excitatory-inhibitory imbalance and genetic epilepsy and febrile seizures plus. *J Neurosci* **2015,** 35, (23), 8866-81.
28. Gupta, A. R.; Westphal, A.; Yang, D. Y. J.; Sullivan, C. A. W.; Eilbott, J.; Zaidi, S.; Voos, A.; Vander Wyk, B. C.; Ventola, P.; Waqar, Z.; Fernandez, T. V.; Ercan-Sencicek, A. G.; Walker, M. F.; Choi, M.; Schneider, A.; Hedderly, T.; Baird, G.; Friedman, H.; Cordeaux, C.; Ristow, A.; Shic, F.; Volkmar, F. R.; Pelphrey, K. A., Neurogenetic analysis of childhood disintegrative disorder. *Mol Autism* **2017,** 8, 19.
29. Ni, Y.; Seballos, S.; Fletcher, B.; Romigh, T.; Yehia, L.; Mester, J.; Senter, L.; Niazi, F.; Saji, M.; Ringel, M. D.; LaFramboise, T.; Eng, C., Germline compound heterozygous poly-glutamine deletion in USF3 may be involved in predisposition to heritable and sporadic epithelial thyroid carcinoma. *Hum Mol Genet* **2017,** 26, (2), 243-257.
30. Monies, D.; Abouelhoda, M.; Assoum, M.; Moghrabi, N.; Rafiullah, R.; Almontashiri, N.; Alowain, M.; Alzaidan, H.; Alsayed, M.; Subhani, S.; Cupler, E.; Faden, M.; Alhashem, A.; Qari, A.; Chedrawi, A.; Aldhalaan, H.; Kurdi, W.; Khan, S.; Rahbeeni, Z.; Alotaibi, M.; Goljan, E.; Elbardisy, H.; ElKalioby, M.; Shah, Z.; Alruwaili, H.; Jaafar, A.; Albar, R.; Akilan, A.; Tayeb, H.; Tahir, A.; Fawzy, M.; Nasr, M.; Makki, S.; Alfaifi, A.; Akleh, H.; Yamani, S.; Bubshait, D.; Mahnashi, M.; Basha, T.; Alsagheir, A.; Khaled, M. A.; Alsaleem, K.; Almugbel, M.; Badawi, M.; Bashiri, F.; Bohlega, S.; Sulaiman, R.; Tous, E.; Ahmed, S.; Algoufi, T.; Al-Mousa, H.; Alaki, E.; Alhumaidi, S.; Alghamdi, H.; Alghamdi, M.; Sahly, A.; Nahrir, S.; Al-Ahmari, A.; Alkuraya, H.; Almehaidib, A.; Abanemai, M.; Alsohaibaini, F.; Alsaud, B.; Arnaout, R.; Abdel-Salam, G. M. H.; Aldhekri, H.; AlKhater, S.; Alqadi, K.; Alsabban, E.; Alshareef, T.; Awartani, K.; Banjar, H.; Alsahan, N.; Abosoudah, I.; Alashwal, A.; Aldekhail, W.; Alhajjar, S.; Al-Mayouf, S.; Alsemari, A.; Alshuaibi, W.; Altala, S.; Altalhi, A.; Baz, S.; Hamad, M.; Abalkhail, T.; Alenazi, B.; Alkaff, A.; Almohareb, F.; Al Mutairi, F.; Alsaleh, M.; Alsonbul, A.; Alzelaye, S.; Bahzad, S.; Manee, A. B.; Jarrad, O.; Meriki, N.; Albeirouti, B.; Alqasmi, A.; AlBalwi, M.; Makhseed, N.; Hassan, S.; Salih, I.; Salih, M. A.; Shaheen, M.; Sermin, S.; Shahrukh, S.; Hashmi, S.; Shawli, A.; Tajuddin, A.; Tamim, A.; Alnahari, A.; Ghemlas, I.; Hussein, M.; Wali,

S.; Murad, H.; Meyer, B. F.; Alkuraya, F. S., Lessons Learned from Large-Scale, First-Tier Clinical Exome Sequencing in a Highly Consanguineous Population. *Am J Hum Genet* **2019,** 105, (4), 879.

31. Fromer, M.; Pocklington, A. J.; Kavanagh, D. H.; Williams, H. J.; Dwyer, S.; Gormley, P.; Georgieva, L.; Rees, E.; Palta, P.; Ruderfer, D. M.; Carrera, N.; Humphreys, I.; Johnson, J. S.; Roussos, P.; Barker, D. D.; Banks, E.; Milanova, V.; Grant, S. G.; Hannon, E.; Rose, S. A.; Chambert, K.; Mahajan, M.; Scolnick, E. M.; Moran, J. L.; Kirov, G.; Palotie, A.; McCarroll, S. A.; Holmans, P.; Sklar, P.; Owen, M. J.; Purcell, S. M.; O'Donovan, M. C., De novo mutations in schizophrenia implicate synaptic networks. *Nature* **2014,** 506, (7487), 179-84.

32. Salpietro, V.; Dixon, C. L.; Guo, H.; Bello, O. D.; Vandrovcova, J.; Efthymiou, S.; Maroofian, R.; Heimer, G.; Burglen, L.; Valence, S.; Torti, E.; Hacke, M.; Rankin, J.; Tariq, H.; Colin, E.; Procaccio, V.; Striano, P.; Mankad, K.; Lieb, A.; Chen, S.; Pisani, L.; Bettencourt, C.; Mannikko, R.; Manole, A.; Brusco, A.; Grosso, E.; Ferrero, G. B.; Armstrong-Moron, J.; Gueden, S.; Bar-Yosef, O.; Tzadok, M.; Monaghan, K. G.; Santiago-Sim, T.; Person, R. E.; Cho, M. T.; Willaert, R.; Yoo, Y.; Chae, J. H.; Quan, Y.; Wu, H.; Wang, T.; Bernier, R. A.; Xia, K.; Blesson, A.; Jain, M.; Motazacker, M. M.; Jaeger, B.; Schneider, A. L.; Boysen, K.; Muir, A. M.; Myers, C. T.; Gavrilova, R. H.; Gunderson, L.; Schultz-Rogers, L.; Klee, E. W.; Dyment, D.; Osmond, M.; Parellada, M.; Llorente, C.; Gonzalez-Penas, J.; Carracedo, A.; Van Haeringen, A.; Ruivenkamp, C.; Nava, C.; Heron, D.; Nardello, R.; Iacomino, M.; Minetti, C.; Skabar, A.; Fabretto, A.; Group, S. S.; Raspall-Chaure, M.; Chez, M.; Tsai, A.; Fassi, E.; Shinawi, M.; Constantino, J. N.; De Zorzi, R.; Fortuna, S.; Kok, F.; Keren, B.; Bonneau, D.; Choi, M.; Benzeev, B.; Zara, F.; Mefford, H. C.; Scheffer, I. E.; Clayton-Smith, J.; Macaya, A.; Rothman, J. E.; Eichler, E. E.; Kullmann, D. M.; Houlden, H., AMPA receptor GluA2 subunit defects are a cause of neurodevelopmental disorders. *Nat Commun* **2019,** 10, (1), 3094.

33. Lin, K. H.; Hu, T. M.; Hsu, S. H.; Tsai, H. Y.; Cheng, M. C., Identification of rare missense mutations in the glutamate ionotropic receptor AMPA type subunit genes in schizophrenia. *Psychiatr Genet* **2023,** 33, (1), 20-25.

34. Hackmann, K.; Matko, S.; Gerlach, E. M.; von der Hagen, M.; Klink, B.; Schrock, E.; Rump, A.; Di Donato, N., Partial deletion of GLRB and GRIA2 in a patient with intellectual disability. *Eur J Hum Genet* **2013,** 21, (1), 112-4.

35. Satterstrom, F. K.; Kosmicki, J. A.; Wang, J.; Breen, M. S.; De Rubeis, S.; An, J. Y.; Peng, M.; Collins, R.; Grove, J.; Klei, L.; Stevens, C.; Reichert, J.; Mulhern, M. S.; Artomov, M.; Gerges, S.; Sheppard, B.; Xu, X.; Bhaduri, A.; Norman, U.; Brand, H.; Schwartz, G.; Nguyen, R.; Guerrero, E. E.; Dias, C.; Autism Sequencing, C.; i, P.-B. C.; Betancur, C.; Cook, E. H.; Gallagher, L.; Gill, M.; Sutcliffe, J. S.; Thurm, A.; Zwick, M. E.; Borglum, A. D.; State, M. W.; Cicek, A. E.; Talkowski, M. E.; Cutler, D. J.; Devlin, B.; Sanders, S. J.; Roeder, K.; Daly, M. J.; Buxbaum, J. D., Large-Scale Exome Sequencing Study Implicates Both Developmental and Functional Changes in the Neurobiology of Autism. *Cell* **2020,** 180, (3), 568-584 e23.

36. Wang, T.; Guo, H.; Xiong, B.; Stessman, H. A.; Wu, H.; Coe, B. P.; Turner, T. N.; Liu, Y.; Zhao, W.; Hoekzema, K.; Vives, L.; Xia, L.; Tang, M.; Ou, J.; Chen, B.; Shen, Y.; Xun, G.; Long, M.; Lin, J.; Kronenberg, Z. N.; Peng, Y.; Bai, T.; Li, H.; Ke, X.; Hu, Z.; Zhao, J.; Zou, X.; Xia, K.; Eichler, E. E., De novo genic mutations among a Chinese autism spectrum disorder cohort. *Nat Commun* **2016,** 7, 13316.

37. Di Gregorio, E.; Riberi, E.; Belligni, E. F.; Biamino, E.; Spielmann, M.; Ala, U.; Calcia, A.; Bagnasco, I.; Carli, D.; Gai, G.; Giordano, M.; Guala, A.; Keller, R.; Mandrile, G.; Arduino, C.; Maffe, A.; Naretto, V. G.; Sirchia, F.; Sorasio, L.; Ungari, S.; Zonta, A.; Zacchetti, G.; Talarico, F.; Pappi, P.; Cavalieri, S.; Giorgio, E.; Mancini, C.; Ferrero, M.; Brussino, A.; Savin, E.; Gandione, M.; Pelle, A.; Giachino, D. F.; De Marchi, M.; Restagno, G.; Provero, P.; Cirillo Silengo, M.; Grosso, E.; Buxbaum, J. D.; Pasini, B.; De Rubeis, S.; Brusco, A.; Ferrero, G. B., Copy number variants analysis in a cohort of isolated and syndromic developmental delay/intellectual disability reveals novel genomic disorders, position effects and candidate disease genes. *Clin Genet* **2017,** 92, (4), 415-422.

38. Zhou, B.; Zhang, C.; Zheng, L.; Wang, Z.; Chen, X.; Feng, X.; Zhang, Q.; Hao, S.; Wei, L.; Gu, W.; Hui, L., Case Report: A Novel De Novo Missense Mutation of the GRIA2 Gene in a Chinese Case of Neurodevelopmental Disorder With Language Impairment. *Front Genet* **2021,** 12, 794766.

39. Coutelier, M.; Jacoupy, M.; Janer, A.; Renaud, F.; Auger, N.; Saripella, G. V.; Ancien, F.; Pucci, F.; Rooman, M.; Gilis, D.; Lariviere, R.; Sgarioto, N.; Valter, R.; Guillot-Noel, L.; Le Ber, I.; Sayah, S.; Charles, P.; Numann, A.; Pauly, M. G.; Helmchen, C.; Deininger, N.; Haack, T. B.; Brais, B.; Brice, A.; Tregouet, D. A.; El Hachimi, K. H.; Shoubridge, E. A.; Durr, A.; Stevanin, G., NPTX1 mutations trigger endoplasmic reticulum stress and cause autosomal dominant cerebellar ataxia. *Brain* **2022,** 145, (4), 1519-1534.

40. Deppe, J.; Deininger, N.; Lingor, P.; Haack, T. B.; Haslinger, B.; Deschauer, M., A Novel NPTX1 de novo Variant in a Late-Onset Ataxia Patient. *Mov Disord* **2022,** 37, (6), 1319-1321.
41. Helmchen, C.; Koch, P. J.; Girard, G.; Bruggemann, N.; Machner, B.; Sprenger, A., NPTX1-related oculomotor apraxia: an intra-hemispheric disconnection disorder. *J Neurol* **2022,** 269, (7), 3931-3936.
42. Schoggl, J.; Siegert, S.; Boltshauser, E.; Freilinger, M.; Schmidt, W. M., A De Novo Missense NPTX1 Variant in an Individual with Infantile-Onset Cerebellar Ataxia. *Mov Disord* **2022,** 37, (8), 1774-1776.
43. Barel, O.; Shalev, S. A.; Ofir, R.; Cohen, A.; Zlotogora, J.; Shorer, Z.; Mazor, G.; Finer, G.; Khateeb, S.; Zilberberg, N.; Birk, O. S., Maternally inherited Birk Barel mental retardation dysmorphism syndrome caused by a mutation in the genomically imprinted potassium channel KCNK9. *Am J Hum Genet* **2008,** 83, (2), 193-9.
44. Graham, J. M., Jr.; Zadeh, N.; Kelley, M.; Tan, E. S.; Liew, W.; Tan, V.; Deardorff, M. A.; Wilson, G. N.; Sagi-Dain, L.; Shalev, S. A., KCNK9 imprinting syndrome-further delineation of a possible treatable disorder. *Am J Med Genet A* **2016,** 170, (10), 2632-7.
45. Sediva, M.; Lassuthova, P.; Zamecnik, J.; Sedlackova, L.; Seeman, P.; Haberlova, J., Novel variant in the KCNK9 gene in a girl with Birk Barel syndrome. *Eur J Med Genet* **2020,** 63, (1), 103619.
46. Klee, E. W.; Cousin, M. A.; Pinto, E. V. F.; Morales-Rosado, J. A.; Macke, E. L.; Jenkinson, W. G.; Ferrer, A.; Schultz-Rogers, L. E.; Olson, R. J.; Oliver, G. R.; Sigafoos, A. N.; Schwab, T. L.; Zimmermann, M. T.; Urrutia, R. A.; Kaiwar, C.; Gupta, A.; Blackburn, P. R.; Boczek, N. J.; Prochnow, C. A.; Lowy, R. J.; Mulvihill, L. A.; McAllister, T. M.; Aoudia, S. L.; Kruisselbrink, T. M.; Gunderson, L. B.; Kemppainen, J. L.; Fisher, L. J.; Tarnowski, J. M.; Hager, M. M.; Kroc, S. A.; Bertsch, N. L.; Agre, K. E.; Jackson, J. L.; Macklin-Mantia, S. K.; Murphree, M. I.; Rust, L. M.; Summer Bolster, J. M.; Beck, S. A.; Atwal, P. S.; Ellingson, M. S.; Barnett, S. S.; Rasmussen, K. J.; Lahner, C. A.; Niu, Z.; Hasadsri, L.; Ferber, M. J.; Marcou, C. A.; Clark, K. J.; Pichurin, P. N.; Deyle, D. R.; Morava-Kozicz, E.; Gavrilova, R. H.; Dhamija, R.; Wierenga, K. J.; Lanpher, B. C.; Babovic-Vuksanovic, D.; Farrugia, G.; Schimmenti, L. A.; Stewart, A. K.; Lazaridis, K. N., Impact of integrated translational research on clinical exome sequencing. *Genet Med* **2021,** 23, (3), 498-507.
47. Yamada, M.; Suzuki, H.; Watanabe, A.; Uehara, T.; Takenouchi, T.; Mizuno, S.; Kosaki, K., Role of chimeric transcript formation in the pathogenesis of birth defects. *Congenit Anom (Kyoto)* **2021,** 61, (3), 76-81.
48. Cousin, M. A.; Veale, E. L.; Dsouza, N. R.; Tripathi, S.; Holden, R. G.; Arelin, M.; Beek, G.; Bekheirnia, M. R.; Beygo, J.; Bhambhani, V.; Bialer, M.; Bigoni, S.; Boelman, C.; Carmichael, J.; Courtin, T.; Cogne, B.; Dabaj, I.; Doummar, D.; Fazilleau, L.; Ferlini, A.; Gavrilova, R. H.; Graham, J. M., Jr.; Haack, T. B.; Juusola, J.; Kant, S. G.; Kayani, S.; Keren, B.; Ketteler, P.; Klockner, C.; Koopmann, T. T.; Kruisselbrink, T. M.; Kuechler, A.; Lambert, L.; Latypova, X.; Lebel, R. R.; Leduc, M. S.; Leonardi, E.; Lewis, A. M.; Liew, W.; Machol, K.; Mardini, S.; McWalter, K.; Mignot, C.; McLaughlin, J.; Murgia, A.; Narayanan, V.; Nava, C.; Neuser, S.; Nizon, M.; Ognibene, D.; Park, J.; Platzer, K.; Poirsier, C.; Radtke, M.; Ramsey, K.; Runke, C. K.; Guillen Sacoto, M. J.; Scaglia, F.; Shinawi, M.; Spranger, S.; Tan, E. S.; Taylor, J.; Trentesaux, A. S.; Vairo, F.; Willaert, R.; Zadeh, N.; Urrutia, R.; Babovic-Vuksanovic, D.; Zimmermann, M. T.; Mathie, A.; Klee, E. W., Gain and loss of TASK3 channel function and its regulation by novel variation cause KCNK9 imprinting syndrome. *Genome Med* **2022,** 14, (1), 62.
49. Guo, H.; Duyzend, M. H.; Coe, B. P.; Baker, C.; Hoekzema, K.; Gerdts, J.; Turner, T. N.; Zody, M. C.; Beighley, J. S.; Murali, S. C.; Nelson, B. J.; University of Washington Center for Mendelian, G.; Bamshad, M. J.; Nickerson, D. A.; Bernier, R. A.; Eichler, E. E., Genome sequencing identifies multiple deleterious variants in autism patients with more severe phenotypes. *Genet Med* **2019,** 21, (7), 1611-1620.
50. Zarrei, M.; Fehlings, D. L.; Mawjee, K.; Switzer, L.; Thiruvahindrapuram, B.; Walker, S.; Merico, D.; Casallo, G.; Uddin, M.; MacDonald, J. R.; Gazzellone, M. J.; Higginbotham, E. J.; Campbell, C.; deVeber, G.; Frid, P.; Gorter, J. W.; Hunt, C.; Kawamura, A.; Kim, M.; McCormick, A.; Mesterman, R.; Samdup, D.; Marshall, C. R.; Stavropoulos, D. J.; Wintle, R. F.; Scherer, S. W., De novo and rare inherited copy-number variations in the hemiplegic form of cerebral palsy. *Genet Med* **2018,** 20, (2), 172-180.
51. Truty, R.; Patil, N.; Sankar, R.; Sullivan, J.; Millichap, J.; Carvill, G.; Entezam, A.; Esplin, E. D.; Fuller, A.; Hogue, M.; Johnson, B.; Khouzam, A.; Kobayashi, Y.; Lewis, R.; Nykamp, K.; Riethmaier, D.; Westbrook, J.; Zeman, M.; Nussbaum, R. L.; Aradhya, S., Possible precision medicine implications from genetic testing using combined detection of sequence and intragenic copy number variants in a large cohort with childhood epilepsy. *Epilepsia Open* **2019,** 4, (3), 397-408.

52. Ganapathy, A.; Mishra, A.; Soni, M. R.; Kumar, P.; Sadagopan, M.; Kanthi, A. V.; Patric, I. R. P.; George, S.; Sridharan, A.; Thyagarajan, T. C.; Aswathy, S. L.; Vidya, H. K.; Chinnappa, S. M.; Nayanala, S.; Prakash, M. B.; Raghavendrachar, V. G.; Parulekar, M.; Gowda, V. K.; Nampoothiri, S.; Menon, R. N.; Pachat, D.; Udani, V.; Naik, N.; Kamate, M.; Devi, A. R. R.; Mohammed Kunju, P. A.; Nair, M.; Hegde, A. U.; Kumar, M. P.; Sundaram, S.; Tilak, P.; Puri, R. D.; Shah, K.; Sheth, J.; Hasan, Q.; Sheth, F.; Agrawal, P.; Katragadda, S.; Veeramachaneni, V.; Chandru, V.; Hariharan, R.; Mannan, A. U., Multi-gene testing in neurological disorders showed an improved diagnostic yield: data from over 1000 Indian patients. *J Neurol* **2019,** 266, (8), 1919-1926.
53. Liu, Y.; Liu, X.; Qin, D.; Zhao, Y.; Cao, X.; Deng, X.; Cheng, Y.; Liu, F.; Yang, F.; Zhang, T.; Yang, X. A., Clinical Utility of Next-Generation Sequencing for Developmental Disorders in the Rehabilitation Department: Experiences from a Single Chinese Center. *J Mol Neurosci* **2021,** 71, (4), 845-853.
54. Wang, T.; Hoekzema, K.; Vecchio, D.; Wu, H.; Sulovari, A.; Coe, B. P.; Gillentine, M. A.; Wilfert, A. B.; Perez-Jurado, L. A.; Kvarnung, M.; Sleyp, Y.; Earl, R. K.; Rosenfeld, J. A.; Geisheker, M. R.; Han, L.; Du, B.; Barnett, C.; Thompson, E.; Shaw, M.; Carroll, R.; Friend, K.; Catford, R.; Palmer, E. E.; Zou, X.; Ou, J.; Li, H.; Guo, H.; Gerdts, J.; Avola, E.; Calabrese, G.; Elia, M.; Greco, D.; Lindstrand, A.; Nordgren, A.; Anderlid, B. M.; Vandeweyer, G.; Van Dijck, A.; Van der Aa, N.; McKenna, B.; Hancarova, M.; Bendova, S.; Havlovicova, M.; Malerba, G.; Bernardina, B. D.; Muglia, P.; van Haeringen, A.; Hoffer, M. J. V.; Franke, B.; Cappuccio, G.; Delatycki, M.; Lockhart, P. J.; Manning, M. A.; Liu, P.; Scheffer, I. E.; Brunetti-Pierri, N.; Rommelse, N.; Amaral, D. G.; Santen, G. W. E.; Trabetti, E.; Sedlacek, Z.; Michaelson, J. J.; Pierce, K.; Courchesne, E.; Kooy, R. F.; Consortium, S.; Nordenskjold, M.; Romano, C.; Peeters, H.; Bernier, R. A.; Gecz, J.; Xia, K.; Eichler, E. E., Author Correction: Large-scale targeted sequencing identifies risk genes for neurodevelopmental disorders. *Nat Commun* **2020,** 11, (1), 5398.
55. Alrakaf, L.; Al-Owain, M. A.; Busehail, M.; Alotaibi, M. A.; Monies, D.; Aldhalaan, H. M.; Alhashem, A.; Al-Hassnan, Z. N.; Rahbeeni, Z. A.; Murshedi, F. A.; Ani, N. A.; Al-Maawali, A.; Ibrahim, N. A.; Abdulwahab, F. M.; Alsagob, M.; Hashem, M. O.; Ramadan, W.; Abouelhoda, M.; Meyer, B. F.; Kaya, N.; Maddirevula, S.; Alkuraya, F. S., Further delineation of Temtamy syndrome of corpus callosum and ocular abnormalities. *Am J Med Genet A* **2018,** 176, (3), 715-721.
56. Akizu, N.; Shembesh, N. M.; Ben-Omran, T.; Bastaki, L.; Al-Tawari, A.; Zaki, M. S.; Koul, R.; Spencer, E.; Rosti, R. O.; Scott, E.; Nickerson, E.; Gabriel, S.; da Gente, G.; Li, J.; Deardorff, M. A.; Conlin, L. K.; Horton, M. A.; Zackai, E. H.; Sherr, E. H.; Gleeson, J. G., Whole-exome sequencing identifies mutated c12orf57 in recessive corpus callosum hypoplasia. *Am J Hum Genet* **2013,** 92, (3), 392-400.
57. Froukh, T. J., Next Generation Sequencing and Genome-Wide Genotyping Identify the Genetic Causes of Intellectual Disability in Ten Consanguineous Families from Jordan. *Tohoku J Exp Med* **2017,** 243, (4), 297-309.
58. Najmabadi, H.; Hu, H.; Garshasbi, M.; Zemojtel, T.; Abedini, S. S.; Chen, W.; Hosseini, M.; Behjati, F.; Haas, S.; Jamali, P.; Zecha, A.; Mohseni, M.; Puttmann, L.; Vahid, L. N.; Jensen, C.; Moheb, L. A.; Bienek, M.; Larti, F.; Mueller, I.; Weissmann, R.; Darvish, H.; Wrogemann, K.; Hadavi, V.; Lipkowitz, B.; Esmaeeli-Nieh, S.; Wieczorek, D.; Kariminejad, R.; Firouzabadi, S. G.; Cohen, M.; Fattahi, Z.; Rost, I.; Mojahedi, F.; Hertzberg, C.; Dehghan, A.; Rajab, A.; Banavandi, M. J.; Hoffer, J.; Falah, M.; Musante, L.; Kalscheuer, V.; Ullmann, R.; Kuss, A. W.; Tzschach, A.; Kahrizi, K.; Ropers, H. H., Deep sequencing reveals 50 novel genes for recessive cognitive disorders. *Nature* **2011,** 478, (7367), 57-63.
59. Charng, W. L.; Karaca, E.; Coban Akdemir, Z.; Gambin, T.; Atik, M. M.; Gu, S.; Posey, J. E.; Jhangiani, S. N.; Muzny, D. M.; Doddapaneni, H.; Hu, J.; Boerwinkle, E.; Gibbs, R. A.; Rosenfeld, J. A.; Cui, H.; Xia, F.; Manickam, K.; Yang, Y.; Faqeih, E. A.; Al Asmari, A.; Saleh, M. A.; El-Hattab, A. W.; Lupski, J. R., Exome sequencing in mostly consanguineous Arab families with neurologic disease provides a high potential molecular diagnosis rate. *BMC Med Genomics* **2016,** 9, (1), 42.
60. Monies, D.; Abouelhoda, M.; AlSayed, M.; Alhassnan, Z.; Alotaibi, M.; Kayyali, H.; Al-Owain, M.; Shah, A.; Rahbeeni, Z.; Al-Muhaizea, M. A.; Alzaidan, H. I.; Cupler, E.; Bohlega, S.; Faqeih, E.; Faden, M.; Alyounes, B.; Jaroudi, D.; Goljan, E.; Elbardisy, H.; Akilan, A.; Albar, R.; Aldhalaan, H.; Gulab, S.; Chedrawi, A.; Al Saud, B. K.; Kurdi, W.; Makhseed, N.; Alqasim, T.; El Khashab, H. Y.; Al-Mousa, H.; Alhashem, A.; Kanaan, I.; Algoufi, T.; Alsaleem, K.; Basha, T. A.; Al-Murshedi, F.; Khan, S.; Al-Kindy, A.; Alnemer, M.; Al-Hajjar, S.; Alyamani, S.; Aldhekri, H.; Al-Mehaidib, A.; Arnaout, R.; Dabbagh, O.; Shagrani, M.; Broering, D.; Tulbah, M.; Alqassmi, A.; Almugbel, M.; AlQuaiz, M.; Alsaman, A.; Al-Thihli, K.; Sulaiman, R. A.; Al-Dekhail, W.; Alsaegh, A.; Bashiri, F. A.; Qari, A.; Alhomadi, S.; Alkuraya, H.; Alsebayel, M.; Hamad, M. H.; Szonyi, L.;

Abaalkhail, F.; Al-Mayouf, S. M.; Almojalli, H.; Alqadi, K. S.; Elsiesy, H.; Shuaib, T. M.; Seidahmed, M. Z.; Abosoudah, I.; Akleh, H.; AlGhonaium, A.; Alkharfy, T. M.; Al Mutairi, F.; Eyaid, W.; Alshanbary, A.; Sheikh, F. R.; Alsohaibani, F. I.; Alsonbul, A.; Al Tala, S.; Balkhy, S.; Bassiouni, R.; Alenizi, A. S.; Hussein, M. H.; Hassan, S.; Khalil, M.; Tabarki, B.; Alshahwan, S.; Oshi, A.; Sabr, Y.; Alsaadoun, S.; Salih, M. A.; Mohamed, S.; Sultana, H.; Tamim, A.; El-Haj, M.; Alshahrani, S.; Bubshait, D. K.; Alfadhel, M.; Faquih, T.; El-Kalioby, M.; Subhani, S.; Shah, Z.; Moghrabi, N.; Meyer, B. F.; Alkuraya, F. S., The landscape of genetic diseases in Saudi Arabia based on the first 1000 diagnostic panels and exomes. *Hum Genet* **2017,** 136, (8), 921-939.

61. Hou, Y. C.; Yu, H. C.; Martin, R.; Cirulli, E. T.; Schenker-Ahmed, N. M.; Hicks, M.; Cohen, I. V.; Jonsson, T. J.; Heister, R.; Napier, L.; Swisher, C. L.; Dominguez, S.; Tang, H.; Li, W.; Perkins, B. A.; Barea, J.; Rybak, C.; Smith, E.; Duchicela, K.; Doney, M.; Brar, P.; Hernandez, N.; Kirkness, E. F.; Kahn, A. M.; Venter, J. C.; Karow, D. S.; Caskey, C. T., Precision medicine integrating whole-genome sequencing, comprehensive metabolomics, and advanced imaging. *Proc Natl Acad Sci U S A* **2020,** 117, (6), 3053-3062.
62. Platzer, K.; Huning, I.; Obieglo, C.; Schwarzmayr, T.; Gabriel, R.; Strom, T. M.; Gillessen-Kaesbach, G.; Kaiser, F. J., Exome sequencing identifies compound heterozygous mutations in C12orf57 in two siblings with severe intellectual disability, hypoplasia of the corpus callosum, chorioretinal coloboma, and intractable seizures. *Am J Med Genet A* **2014,** 164A, (8), 1976-80.
63. Gronskov, K.; Brondum-Nielsen, K.; Dedic, A.; Hjalgrim, H., A nonsense mutation in FMR1 causing fragile X syndrome. *Eur J Hum Genet* **2011,** 19, (4), 489-91.
64. Collins, S. C.; Bray, S. M.; Suhl, J. A.; Cutler, D. J.; Coffee, B.; Zwick, M. E.; Warren, S. T., Identification of novel FMR1 variants by massively parallel sequencing in developmentally delayed males. *Am J Med Genet A* **2010,** 152A, (10), 2512-20.
65. De Boulle, K.; Verkerk, A. J.; Reyniers, E.; Vits, L.; Hendrickx, J.; Van Roy, B.; Van den Bos, F.; de Graaff, E.; Oostra, B. A.; Willems, P. J., A point mutation in the FMR-1 gene associated with fragile X mental retardation. *Nat Genet* **1993,** 3, (1), 31-5.
66. Handt, M.; Epplen, A.; Hoffjan, S.; Mese, K.; Epplen, J. T.; Dekomien, G., Point mutation frequency in the FMR1 gene as revealed by fragile X syndrome screening. *Mol Cell Probes* **2014,** 28, (5-6), 279-83.
67. Quartier, A.; Poquet, H.; Gilbert-Dussardier, B.; Rossi, M.; Casteleyn, A. S.; Portes, V. D.; Feger, C.; Nourisson, E.; Kuentz, P.; Redin, C.; Thevenon, J.; Mosca-Boidron, A. L.; Callier, P.; Muller, J.; Lesca, G.; Huet, F.; Geoffroy, V.; El Chehadeh, S.; Jung, M.; Trojak, B.; Le Gras, S.; Lehalle, D.; Jost, B.; Maury, S.; Masurel, A.; Edery, P.; Thauvin-Robinet, C.; Gerard, B.; Mandel, J. L.; Faivre, L.; Piton, A., Intragenic FMR1 disease-causing variants: a significant mutational mechanism leading to Fragile-X syndrome. *Eur J Hum Genet* **2017,** 25, (4), 423-431.
68. Ibarluzea, N.; Hoz, A. B.; Villate, O.; Llano, I.; Ocio, I.; Marti, I.; Guitart, M.; Gabau, E.; Andrade, F.; Gener, B.; Tejada, M. I., Targeted Next-Generation Sequencing in Patients with Suggestive X-Linked Intellectual Disability. *Genes (Basel)* **2020,** 11, (1).
69. Maddirevula, S.; Alsaif, H. S.; Ibrahim, N.; Alkuraya, F. S., A de novo mutation in FMR1 in a patient with intellectual disability. *Eur J Med Genet* **2020,** 63, (3), 103763.
70. Pereira, R. R.; Pinto, I. P.; Minasi, L. B.; de Melo, A. V.; da Cruz e Cunha, D. M.; Cruz, A. S.; Ribeiro, C. L.; da Silva, C. C.; de Melo e Silva, D.; da Cruz, A. D., Screening for intellectual disability using high-resolution CMA technology in a retrospective cohort from Central Brazil. *PLoS One* **2014,** 9, (7), e103117.
71. Jorge, P.; Oliveira, B.; Marques, I.; Santos, R., Development and validation of a multiplex-PCR assay for X-linked intellectual disability. *BMC Med Genet* **2013,** 14, 80.
72. Nagamani, S. C.; Erez, A.; Probst, F. J.; Bader, P.; Evans, P.; Baker, L. A.; Fang, P.; Bertin, T.; Hixson, P.; Stankiewicz, P.; Nelson, D.; Patel, A.; Cheung, S. W., Small genomic rearrangements involving FMR1 support the importance of its gene dosage for normal neurocognitive function. *Neurogenetics* **2012,** 13, (4), 333-9.
73. Zink, A. M.; Wohlleber, E.; Engels, H.; Rodningen, O. K.; Ravn, K.; Heilmann, S.; Rehnitz, J.; Katzorke, N.; Kraus, C.; Blichfeldt, S.; Hoffmann, P.; Reutter, H.; Brockschmidt, F. F.; Kreiss-Nachtsheim, M.; Vogt, P. H.; Prescott, T. E.; Tumer, Z.; Lee, J. A., Microdeletions including FMR1 in three female patients with intellectual disability - further delineation of the phenotype and expression studies. *Mol Syndromol* **2014,** 5, (2), 65-75.

74. Chaves, T. F.; Baretto, N.; Oliveira, L. F.; Ocampos, M.; Barbato, I. T.; Anselmi, M.; De Luca, G. R.; Barbato Filho, J. H.; Pinto, L. L. C.; Bernardi, P.; Maris, A. F., Copy Number Variations in a Cohort of 420 Individuals with Neurodevelopmental Disorders From the South of Brazil. *Sci Rep* **2019,** 9, (1), 17776.
75. Trost, B.; Engchuan, W.; Nguyen, C. M.; Thiruvahindrapuram, B.; Dolzhenko, E.; Backstrom, I.; Mirceta, M.; Mojarad, B. A.; Yin, Y.; Dov, A.; Chandrakumar, I.; Prasolava, T.; Shum, N.; Hamdan, O.; Pellecchia, G.; Howe, J. L.; Whitney, J.; Klee, E. W.; Baheti, S.; Amaral, D. G.; Anagnostou, E.; Elsabbagh, M.; Fernandez, B. A.; Hoang, N.; Lewis, M. E. S.; Liu, X.; Sjaarda, C.; Smith, I. M.; Szatmari, P.; Zwaigenbaum, L.; Glazer, D.; Hartley, D.; Stewart, A. K.; Eberle, M. A.; Sato, N.; Pearson, C. E.; Scherer, S. W.; Yuen, R. K. C., Genome-wide detection of tandem DNA repeats that are expanded in autism. *Nature* **2020,** 586, (7827), 80-86.
76. Husson, T.; Lecoquierre, F.; Cassinari, K.; Charbonnier, C.; Quenez, O.; Goldenberg, A.; Guerrot, A. M.; Richard, A. C.; Drouin-Garraud, V.; Brehin, A. C.; Soleimani, M.; Taton, R.; Rotharmel, M.; Rosier, A.; Chambon, P.; Le Meur, N.; Joly-Helas, G.; Saugier-Veber, P.; Boland, A.; Deleuze, J. F.; Olaso, R.; Frebourg, T.; Nicolas, G.; Guillin, O.; Campion, D., Rare genetic susceptibility variants assessment in autism spectrum disorder: detection rate and practical use. *Transl Psychiatry* **2020,** 10, (1), 77.
77. Vengoechea, J.; Parikh, A. S.; Zhang, S.; Tassone, F., De novo microduplication of the FMR1 gene in a patient with developmental delay, epilepsy and hyperactivity. *Eur J Hum Genet* **2012,** 20, (11), 1197-200.
78. Myers, K. A.; van 't Hof, F. N. G.; Sadleir, L. G.; Legault, G.; Simard-Tremblay, E.; Amor, D. J.; Scheffer, I. E., Fragile Females: Case Series of Epilepsy in Girls With FMR1 Disruption. *Pediatrics* **2019,** 144, (3).
79. Katoh, K.; Aiba, K.; Fukushi, D.; Yoshimura, J.; Suzuki, Y.; Mitsui, J.; Morishita, S.; Tuji, S.; Yamada, K.; Wakamatsu, N., Clinical and molecular genetic characterization of two female patients harboring the Xq27.3q28 deletion with different ratios of X chromosome inactivation. *Hum Mutat* **2020,** 41, (8), 1447-1460.
80. Loesch, D. Z.; Khaniani, M. S.; Slater, H. R.; Rubio, J. P.; Bui, Q. M.; Kotschet, K.; D'Souza, W.; Venn, A.; Kalitsis, P.; Choo, A. K.; Burgess, T.; Johnson, L.; Evans, A.; Horne, M., Small CGG repeat expansion alleles of FMR1 gene are associated with parkinsonism. *Clin Genet* **2009,** 76, (5), 471-6.
81. Park, K. M.; Jun, K. R.; Lee, H. J.; Park, S.; Kim, S. E., Unilateral diffuse white matter involvement in a patient with atypical FMR1 mutation. *Clin Neurol Neurosurg* **2020,** 197, 106182.
82. Cortes, H. D.; Wevrick, R., Genetic analysis of very obese children with autism spectrum disorder. *Mol Genet Genomics* **2018,** 293, (3), 725-736.
83. Itai, T.; Jia, P.; Dai, Y.; Chen, J.; Chen, X.; Zhao, Z., De novo mutations disturb early brain development more frequently than common variants in schizophrenia. *Am J Med Genet B Neuropsychiatr Genet* **2023,** 192, (3-4), 62-70.
84. Vulto-van Silfhout, A. T.; de Brouwer, A. F.; de Leeuw, N.; Obihara, C. C.; Brunner, H. G.; de Vries, B. B., A 380-kb Duplication in 7p22.3 Encompassing the LFNG Gene in a Boy with Asperger Syndrome. *Mol Syndromol* **2012,** 2, (6), 245-250.
85. Mannucci, I.; Dang, N. D. P.; Huber, H.; Murry, J. B.; Abramson, J.; Althoff, T.; Banka, S.; Baynam, G.; Bearden, D.; Beleza-Meireles, A.; Benke, P. J.; Berland, S.; Bierhals, T.; Bilan, F.; Bindoff, L. A.; Braathen, G. J.; Busk, O. L.; Chenbhanich, J.; Denecke, J.; Escobar, L. F.; Estes, C.; Fleischer, J.; Groepper, D.; Haaxma, C. A.; Hempel, M.; Holler-Managan, Y.; Houge, G.; Jackson, A.; Kellogg, L.; Keren, B.; Kiraly-Borri, C.; Kraus, C.; Kubisch, C.; Le Guyader, G.; Ljungblad, U. W.; Brenman, L. M.; Martinez-Agosto, J. A.; Might, M.; Miller, D. T.; Minks, K. Q.; Moghaddam, B.; Nava, C.; Nelson, S. F.; Parant, J. M.; Prescott, T.; Rajabi, F.; Randrianaivo, H.; Reiter, S. F.; Schuurs-Hoeijmakers, J.; Shieh, P. B.; Slavotinek, A.; Smithson, S.; Stegmann, A. P. A.; Tomczak, K.; Tveten, K.; Wang, J.; Whitlock, J. H.; Zweier, C.; McWalter, K.; Juusola, J.; Quintero-Rivera, F.; Fischer, U.; Yeo, N. C.; Kreienkamp, H. J.; Lessel, D., Genotype-phenotype correlations and novel molecular insights into the DHX30-associated neurodevelopmental disorders. *Genome Med* **2021,** 13, (1), 90.
86. Cross, L. A.; McWalter, K.; Keller-Ramey, J.; Henderson, L. B.; Amudhavalli, S. M., A report of gonadal mosaicism in DHX30-related neurodevelopmental disorder. *Clin Dysmorphol* **2020,** 29, (3), 161-164.
87. Miyake, N.; Kim, C. A.; Haginoya, K.; Castro, M. A. A.; Honjo, R. S.; Matsumoto, N., De novo pathogenic DHX30 variants in two cases. *Clin Genet* **2021,** 100, (3), 350-351.
88. Ueda, K.; Araki, A.; Fujita, A.; Matsumoto, N.; Uehara, T.; Suzuki, H.; Takenouchi, T.; Kosaki, K.; Okamoto, N., A Japanese adult and two girls with NEDMIAL caused by de novo missense variants in DHX30. *Hum Genome Var* **2021,** 8, (1), 24.
89. Lessel, D.; Schob, C.; Kury, S.; Reijnders, M. R. F.; Harel, T.; Eldomery, M. K.; Coban-Akdemir, Z.; Denecke, J.; Edvardson, S.; Colin, E.; Stegmann, A. P. A.; Gerkes, E. H.; Tessarech, M.; Bonneau, D.; Barth, M.;

Besnard, T.; Cogne, B.; Revah-Politi, A.; Strom, T. M.; Rosenfeld, J. A.; Yang, Y.; Posey, J. E.; Immken, L.; Oundjian, N.; Helbig, K. L.; Meeks, N.; Zegar, K.; Morton, J.; study, D. D. D.; Schieving, J. H.; Claasen, A.; Huentelman, M.; Narayanan, V.; Ramsey, K.; Group, C. R. R.; Brunner, H. G.; Elpeleg, O.; Mercier, S.; Bezieau, S.; Kubisch, C.; Kleefstra, T.; Kindler, S.; Lupski, J. R.; Kreienkamp, H. J., De Novo Missense Mutations in DHX30 Impair Global Translation and Cause a Neurodevelopmental Disorder. *Am J Hum Genet* **2017,** 101, (5), 716-724.
90. Deciphering Developmental Disorders, S., Prevalence and architecture of de novo mutations in developmental disorders. *Nature* **2017,** 542, (7642), 433-438.
91. Eldomery, M. K.; Coban-Akdemir, Z.; Harel, T.; Rosenfeld, J. A.; Gambin, T.; Stray-Pedersen, A.; Kury, S.; Mercier, S.; Lessel, D.; Denecke, J.; Wiszniewski, W.; Penney, S.; Liu, P.; Bi, W.; Lalani, S. R.; Schaaf, C. P.; Wangler, M. F.; Bacino, C. A.; Lewis, R. A.; Potocki, L.; Graham, B. H.; Belmont, J. W.; Scaglia, F.; Orange, J. S.; Jhangiani, S. N.; Chiang, T.; Doddapaneni, H.; Hu, J.; Muzny, D. M.; Xia, F.; Beaudet, A. L.; Boerwinkle, E.; Eng, C. M.; Plon, S. E.; Sutton, V. R.; Gibbs, R. A.; Posey, J. E.; Yang, Y.; Lupski, J. R., Lessons learned from additional research analyses of unsolved clinical exome cases. *Genome Med* **2017,** 9, (1), 26.
92. Andrews, A.; Maharaj, A.; Cottrell, E.; Chatterjee, S.; Shah, P.; Denvir, L.; Dumic, K.; Bossowski, A.; Mushtaq, T.; Vukovic, R.; Didi, M.; Shaw, N.; Metherell, L. A.; Savage, M. O.; Storr, H. L., Genetic Characterization of Short Stature Patients With Overlapping Features of Growth Hormone Insensitivity Syndromes. *J Clin Endocrinol Metab* **2021,** 106, (11), e4716-e4733.
93. Basel-Vanagaite, L.; Sarig, O.; Hershkovitz, D.; Fuchs-Telem, D.; Rapaport, D.; Gat, A.; Isman, G.; Shirazi, I.; Shohat, M.; Enk, C. D.; Birk, E.; Kohlhase, J.; Matysiak-Scholze, U.; Maya, I.; Knopf, C.; Peffekoven, A.; Hennies, H. C.; Bergman, R.; Horowitz, M.; Ishida-Yamamoto, A.; Sprecher, E., RIN2 deficiency results in macrocephaly, alopecia, cutis laxa, and scoliosis: MACS syndrome. *Am J Hum Genet* **2009,** 85, (2), 254-63.
94. Syx, D.; Malfait, F.; Van Laer, L.; Hellemans, J.; Hermanns-Le, T.; Willaert, A.; Benmansour, A.; De Paepe, A.; Verloes, A., The RIN2 syndrome: a new autosomal recessive connective tissue disorder caused by deficiency of Ras and Rab interactor 2 (RIN2). *Hum Genet* **2010,** 128, (1), 79-88.
95. Shaukat, M.; Ishaq, T.; Muhammad, N.; Naz, S., RIN2 and BBS7 variants as cause of a coincidental syndrome. *Eur J Med Genet* **2020,** 63, (3), 103755.
96. Kameli, R.; Ashrafi, M. R.; Ehya, F.; Alizadeh, H.; Hosseinpour, S.; Garshasbi, M.; Tavasoli, A. R., Leukoencephalopathy in RIN2 syndrome: Novel mutation and expansion of clinical spectrum. *Eur J Med Genet* **2020,** 63, (1), 103629.
97. Xu, B.; Ionita-Laza, I.; Roos, J. L.; Boone, B.; Woodrick, S.; Sun, Y.; Levy, S.; Gogos, J. A.; Karayiorgou, M., De novo gene mutations highlight patterns of genetic and neural complexity in schizophrenia. *Nat Genet* **2012,** 44, (12), 1365-9.
98. Peng, J.; Wang, Y.; He, F.; Chen, C.; Wu, L. W.; Yang, L. F.; Ma, Y. P.; Zhang, W.; Shi, Z. Q.; Chen, C.; Xia, K.; Guo, H.; Yin, F.; Pang, N., Novel West syndrome candidate genes in a Chinese cohort. *CNS Neurosci Ther* **2018,** 24, (12), 1196-1206.
99. Lim, E. T.; Raychaudhuri, S.; Sanders, S. J.; Stevens, C.; Sabo, A.; MacArthur, D. G.; Neale, B. M.; Kirby, A.; Ruderfer, D. M.; Fromer, M.; Lek, M.; Liu, L.; Flannick, J.; Ripke, S.; Nagaswamy, U.; Muzny, D.; Reid, J. G.; Hawes, A.; Newsham, I.; Wu, Y.; Lewis, L.; Dinh, H.; Gross, S.; Wang, L. S.; Lin, C. F.; Valladares, O.; Gabriel, S. B.; dePristo, M.; Altshuler, D. M.; Purcell, S. M.; Project, N. E. S.; State, M. W.; Boerwinkle, E.; Buxbaum, J. D.; Cook, E. H.; Gibbs, R. A.; Schellenberg, G. D.; Sutcliffe, J. S.; Devlin, B.; Roeder, K.; Daly, M. J., Rare complete knockouts in humans: population distribution and significant role in autism spectrum disorders. *Neuron* **2013,** 77, (2), 235-42.
100. Whibley, A. C.; Plagnol, V.; Tarpey, P. S.; Abidi, F.; Fullston, T.; Choma, M. K.; Boucher, C. A.; Shepherd, L.; Willatt, L.; Parkin, G.; Smith, R.; Futreal, P. A.; Shaw, M.; Boyle, J.; Licata, A.; Skinner, C.; Stevenson, R. E.; Turner, G.; Field, M.; Hackett, A.; Schwartz, C. E.; Gecz, J.; Stratton, M. R.; Raymond, F. L., Fine-scale survey of X chromosome copy number variants and indels underlying intellectual disability. *Am J Hum Genet* **2010,** 87, (2), 173-88.
101. Sirrs, S.; van Karnebeek, C. D.; Peng, X.; Shyr, C.; Tarailo-Graovac, M.; Mandal, R.; Testa, D.; Dubin, D.; Carbonetti, G.; Glynn, S. E.; Sayson, B.; Robinson, W. P.; Han, B.; Wishart, D.; Ross, C. J.; Wasserman, W. W.; Hurwitz, T. A.; Sinclair, G.; Kaczocha, M., Defects in fatty acid amide hydrolase 2 in a male with neurologic and psychiatric symptoms. *Orphanet J Rare Dis* **2015,** 10, 38.

102. Kars, M. E.; Basak, A. N.; Onat, O. E.; Bilguvar, K.; Choi, J.; Itan, Y.; Caglar, C.; Palvadeau, R.; Casanova, J. L.; Cooper, D. N.; Stenson, P. D.; Yavuz, A.; Bulus, H.; Gunel, M.; Friedman, J. M.; Ozcelik, T., The genetic structure of the Turkish population reveals high levels of variation and admixture. *Proc Natl Acad Sci U S A* **2021,** 118, (36).
103. Shamseldin, H. E.; AlAbdi, L.; Maddirevula, S.; Alsaif, H. S.; Alzahrani, F.; Ewida, N.; Hashem, M.; Abdulwahab, F.; Abuyousef, O.; Kuwahara, H.; Gao, X.; Molecular Autopsy, C.; Alkuraya, F. S., Lethal variants in humans: lessons learned from a large molecular autopsy cohort. *Genome Med* **2021,** 13, (1), 161.
104. Johnson, B. V.; Kumar, R.; Oishi, S.; Alexander, S.; Kasherman, M.; Vega, M. S.; Ivancevic, A.; Gardner, A.; Domingo, D.; Corbett, M.; Parnell, E.; Yoon, S.; Oh, T.; Lines, M.; Lefroy, H.; Kini, U.; Van Allen, M.; Gronborg, S.; Mercier, S.; Kury, S.; Bezieau, S.; Pasquier, L.; Raynaud, M.; Afenjar, A.; Billette de Villemeur, T.; Keren, B.; Desir, J.; Van Maldergem, L.; Marangoni, M.; Dikow, N.; Koolen, D. A.; VanHasselt, P. M.; Weiss, M.; Zwijnenburg, P.; Sa, J.; Reis, C. F.; Lopez-Otin, C.; Santiago-Fernandez, O.; Fernandez-Jaen, A.; Rauch, A.; Steindl, K.; Joset, P.; Goldstein, A.; Madan-Khetarpal, S.; Infante, E.; Zackai, E.; McDougall, C.; Narayanan, V.; Ramsey, K.; Mercimek-Andrews, S.; Pena, L.; Shashi, V.; Undiagnosed Diseases, N.; Schoch, K.; Sullivan, J. A.; Pinto, E. V. F.; Pichurin, P. N.; Ewing, S. A.; Barnett, S. S.; Klee, E. W.; Perry, M. S.; Koenig, M. K.; Keegan, C. E.; Schuette, J. L.; Asher, S.; Perilla-Young, Y.; Smith, L. D.; Rosenfeld, J. A.; Bhoj, E.; Kaplan, P.; Li, D.; Oegema, R.; van Binsbergen, E.; van der Zwaag, B.; Smeland, M. F.; Cutcutache, I.; Page, M.; Armstrong, M.; Lin, A. E.; Steeves, M. A.; Hollander, N. D.; Hoffer, M. J. V.; Reijnders, M. R. F.; Demirdas, S.; Koboldt, D. C.; Bartholomew, D.; Mosher, T. M.; Hickey, S. E.; Shieh, C.; Sanchez-Lara, P. A.; Graham, J. M., Jr.; Tezcan, K.; Schaefer, G. B.; Danylchuk, N. R.; Asamoah, A.; Jackson, K. E.; Yachelevich, N.; Au, M.; Perez-Jurado, L. A.; Kleefstra, T.; Penzes, P.; Wood, S. A.; Burne, T.; Pierson, T. M.; Piper, M.; Gecz, J.; Jolly, L. A., Partial Loss of USP9X Function Leads to a Male Neurodevelopmental and Behavioral Disorder Converging on Transforming Growth Factor beta Signaling. *Biol Psychiatry* **2020,** 87, (2), 100-112.
105. Jolly, L. A.; Parnell, E.; Gardner, A. E.; Corbett, M. A.; Perez-Jurado, L. A.; Shaw, M.; Lesca, G.; Keegan, C.; Schneider, M. C.; Griffin, E.; Maier, F.; Kiss, C.; Guerin, A.; Crosby, K.; Rosenbaum, K.; Tanpaiboon, P.; Whalen, S.; Keren, B.; McCarrier, J.; Basel, D.; Sadedin, S.; White, S. M.; Delatycki, M. B.; Kleefstra, T.; Kury, S.; Brusco, A.; Sukarova-Angelovska, E.; Trajkova, S.; Yoon, S.; Wood, S. A.; Piper, M.; Penzes, P.; Gecz, J., Missense variant contribution to USP9X-female syndrome. *NPJ Genom Med* **2020,** 5, (1), 53.
106. Tsurusaki, Y.; Kuroda, Y.; Yamanouchi, Y.; Kondo, E.; Ouchi, K.; Kimura, Y.; Enomoto, Y.; Aida, N.; Masuno, M.; Kurosawa, K., Novel USP9X variants in two patients with X-linked intellectual disability. *Hum Genome Var* **2019,** 6, 49.
107. Al-Mubarak, B.; Abouelhoda, M.; Omar, A.; AlDhalaan, H.; Aldosari, M.; Nester, M.; Alshamrani, H. A.; El-Kalioby, M.; Goljan, E.; Albar, R.; Subhani, S.; Tahir, A.; Asfahani, S.; Eskandrani, A.; Almusaiab, A.; Magrashi, A.; Shinwari, J.; Monies, D.; Al Tassan, N., Whole exome sequencing reveals inherited and de novo variants in autism spectrum disorder: a trio study from Saudi families. *Sci Rep* **2017,** 7, (1), 5679.
108. Paemka, L.; Mahajan, V. B.; Ehaideb, S. N.; Skeie, J. M.; Tan, M. C.; Wu, S.; Cox, A. J.; Sowers, L. P.; Gecz, J.; Jolly, L.; Ferguson, P. J.; Darbro, B.; Schneider, A.; Scheffer, I. E.; Carvill, G. L.; Mefford, H. C.; El-Shanti, H.; Wood, S. A.; Manak, J. R.; Bassuk, A. G., Seizures are regulated by ubiquitin-specific peptidase 9 X-linked (USP9X), a de-ubiquitinase. *PLoS Genet* **2015,** 11, (3), e1005022.
109. Marinakis, N. M.; Svingou, M.; Veltra, D.; Kekou, K.; Sofocleous, C.; Tilemis, F. N.; Kosma, K.; Tsoutsou, E.; Fryssira, H.; Traeger-Synodinos, J., Phenotype-driven variant filtration strategy in exome sequencing toward a high diagnostic yield and identification of 85 novel variants in 400 patients with rare Mendelian disorders. *Am J Med Genet A* **2021,** 185, (8), 2561-2571.
110. Ngo, K. J.; Rexach, J. E.; Lee, H.; Petty, L. E.; Perlman, S.; Valera, J. M.; Deignan, J. L.; Mao, Y.; Aker, M.; Posey, J. E.; Jhangiani, S. N.; Coban-Akdemir, Z. H.; Boerwinkle, E.; Muzny, D.; Nelson, A. B.; Hassin-Baer, S.; Poke, G.; Neas, K.; Geschwind, M. D.; Grody, W. W.; Gibbs, R.; Geschwind, D. H.; Lupski, J. R.; Below, J. E.; Nelson, S. F.; Fogel, B. L., A diagnostic ceiling for exome sequencing in cerebellar ataxia and related neurological disorders. *Hum Mutat* **2020,** 41, (2), 487-501.
111. Jiang, T.; Gao, J.; Jiang, L.; Xu, L.; Zhao, C.; Su, X.; Shen, Y.; Gu, W.; Kong, X.; Yang, Y.; Gao, F., Application of Trio-Whole Exome Sequencing in Genetic Diagnosis and Therapy in Chinese Children With Epilepsy. *Front Mol Neurosci* **2021,** 14, 699574.

112. Genovese, G.; Fromer, M.; Stahl, E. A.; Ruderfer, D. M.; Chambert, K.; Landen, M.; Moran, J. L.; Purcell, S. M.; Sklar, P.; Sullivan, P. F.; Hultman, C. M.; McCarroll, S. A., Increased burden of ultra-rare protein-altering variants among 4,877 individuals with schizophrenia. *Nat Neurosci* **2016,** 19, (11), 1433-1441.
113. Benkirane, M.; Marelli, C.; Guissart, C.; Roubertie, A.; Ollagnon, E.; Choumert, A.; Fluchere, F.; Magne, F. O.; Halleb, Y.; Renaud, M.; Larrieu, L.; Baux, D.; Patat, O.; Bousquet, I.; Ravel, J. M.; Cuntz-Shadfar, D.; Sarret, C.; Ayrignac, X.; Rolland, A.; Morales, R.; Pointaux, M.; Lieutard-Haag, C.; Laurens, B.; Tillikete, C.; Bernard, E.; Mallaret, M.; Carra-Dalliere, C.; Tranchant, C.; Meyer, P.; Damaj, L.; Pasquier, L.; Acquaviva, C.; Chaussenot, A.; Isidor, B.; Nguyen, K.; Camu, W.; Eusebio, A.; Carriere, N.; Riquet, A.; Thouvenot, E.; Gonzales, V.; Carme, E.; Attarian, S.; Odent, S.; Castrioto, A.; Ewenczyk, C.; Charles, P.; Kremer, L.; Sissaoui, S.; Bahi-Buisson, N.; Kaphan, E.; Degardin, A.; Doray, B.; Julia, S.; Remerand, G.; Fraix, V.; Haidar, L. A.; Lazaro, L.; Laugel, V.; Villega, F.; Charlin, C.; Frismand, S.; Moreira, M. C.; Witjas, T.; Francannet, C.; Walther-Louvier, U.; Fradin, M.; Chabrol, B.; Fluss, J.; Bieth, E.; Castelnovo, G.; Vergnet, S.; Meunier, I.; Verloes, A.; Brischoux-Boucher, E.; Coubes, C.; Genevieve, D.; Lebouc, N.; Azulay, J. P.; Anheim, M.; Goizet, C.; Rivier, F.; Labauge, P.; Calvas, P.; Koenig, M., High rate of hypomorphic variants as the cause of inherited ataxia and related diseases: study of a cohort of 366 families. *Genet Med* **2021,** 23, (11), 2160-2170.
114. Jin, S. C.; Lewis, S. A.; Bakhtiari, S.; Zeng, X.; Sierant, M. C.; Shetty, S.; Nordlie, S. M.; Elie, A.; Corbett, M. A.; Norton, B. Y.; van Eyk, C. L.; Haider, S.; Guida, B. S.; Magee, H.; Liu, J.; Pastore, S.; Vincent, J. B.; Brunstrom-Hernandez, J.; Papavasileiou, A.; Fahey, M. C.; Berry, J. G.; Harper, K.; Zhou, C.; Zhang, J.; Li, B.; Zhao, H.; Heim, J.; Webber, D. L.; Frank, M. S. B.; Xia, L.; Xu, Y.; Zhu, D.; Zhang, B.; Sheth, A. H.; Knight, J. R.; Castaldi, C.; Tikhonova, I. R.; Lopez-Giraldez, F.; Keren, B.; Whalen, S.; Buratti, J.; Doummar, D.; Cho, M.; Retterer, K.; Millan, F.; Wang, Y.; Waugh, J. L.; Rodan, L.; Cohen, J. S.; Fatemi, A.; Lin, A. E.; Phillips, J. P.; Feyma, T.; MacLennan, S. C.; Vaughan, S.; Crompton, K. E.; Reid, S. M.; Reddihough, D. S.; Shang, Q.; Gao, C.; Novak, I.; Badawi, N.; Wilson, Y. A.; McIntyre, S. J.; Mane, S. M.; Wang, X.; Amor, D. J.; Zarnescu, D. C.; Lu, Q.; Xing, Q.; Zhu, C.; Bilguvar, K.; Padilla-Lopez, S.; Lifton, R. P.; Gecz, J.; MacLennan, A. H.; Kruer, M. C., Author Correction: Mutations disrupting neuritogenesis genes confer risk for cerebral palsy. *Nat Genet* **2021,** 53, (3), 412.
115. Drielsma, A.; Jalas, C.; Simonis, N.; Desir, J.; Simanovsky, N.; Pirson, I.; Elpeleg, O.; Abramowicz, M.; Edvardson, S., Two novel CCDC88C mutations confirm the role of DAPLE in autosomal recessive congenital hydrocephalus. *J Med Genet* **2012,** 49, (11), 708-12.
116. Ekici, A. B.; Hilfinger, D.; Jatzwauk, M.; Thiel, C. T.; Wenzel, D.; Lorenz, I.; Boltshauser, E.; Goecke, T. W.; Staatz, G.; Morris-Rosendahl, D. J.; Sticht, H.; Hehr, U.; Reis, A.; Rauch, A., Disturbed Wnt Signalling due to a Mutation in CCDC88C Causes an Autosomal Recessive Non-Syndromic Hydrocephalus with Medial Diverticulum. *Mol Syndromol* **2010,** 1, (3), 99-112.
117. Ruggeri, G.; Timms, A. E.; Cheng, C.; Weiss, A.; Kollros, P.; Chapman, T.; Tully, H.; Mirzaa, G. M., Bi-allelic mutations of CCDC88C are a rare cause of severe congenital hydrocephalus. *Am J Med Genet A* **2018,** 176, (3), 676-681.
118. Marguet, F.; Vezain, M.; Marcorelles, P.; Audebert-Bellanger, S.; Cassinari, K.; Drouot, N.; Chambon, P.; Gonzalez, B. J.; Horowitz, A.; Laquerriere, A.; Saugier-Veber, P., Neuropathological hallmarks of fetal hydrocephalus linked to CCDC88C pathogenic variants. *Acta Neuropathol Commun* **2021,** 9, (1), 104.
119. Guo, J. J.; Wang, Z. Y.; Zhang, S.; Wang, D. L.; Tian, L.; Liu, J. Y.; Zhu, H., Paroxysmal limbs jitter accompanied by different imaging findings in a Chinese family with spinocerebellar ataxia 40: Clinical and neuroimaging studies. *Neuro Endocrinol Lett* **2021,** 42, (4), 215-221.
120. Boros, F. A.; Szpisjak, L.; Bozo, R.; Kelemen, E.; Zadori, D.; Salamon, A.; Danis, J.; Kalmar, T.; Maroti, Z.; Molnar, M. J.; Klivenyi, P.; Szell, M.; Adam, E., Spinocerebellar Ataxia in a Hungarian Female Patient with a Novel Variant of Unknown Significance in the CCDC88C Gene. *Int J Mol Sci* **2023,** 24, (3).
121. Perkovic, R.; Hrvoic, L.; Mandic, I.; Koruga, A. S.; Soldo, S. B., Novel mutation for SCA40 with initial presentation as retinitis pigmentosa. *Acta Neurol Belg* **2023,** 123, (5), 2027-2029.
122. Han, F.; Su, D.; Qu, C., Erratum to "Spinocerebellar ataxia type 40: A case report and literature review". *Transl Neurosci* **2022,** 13, (1), 70.
123. Emamikhah, M.; Aghavali, S.; Moghadas, F.; Munhoz, R. P.; Lang, A. E.; Alavi, A.; Rohani, M., Spinocerebellar Ataxia 40: Another Etiology Underlying Essential Tremor Syndrome. *Mov Disord Clin Pract* **2021,** 8, (6), 944-946.

124. Wan, N.; Chen, Z.; Wan, L.; Yuan, H.; Tang, Z.; Liu, M.; Peng, Y.; Peng, L.; Lei, L.; Xie, Y.; Deng, Q.; Wang, S.; Wang, C.; Peng, H.; Hou, X.; Shi, Y.; Long, Z.; Qiu, R.; Xia, K.; Tang, B.; Jiang, H., Genetic etiology of a Chinese ataxia cohort: Expanding the mutational spectrum of hereditary ataxias. *Parkinsonism Relat Disord* **2021,** 89, 120-127.
125. Galatolo, D.; De Michele, G.; Silvestri, G.; Leuzzi, V.; Casali, C.; Musumeci, O.; Antenora, A.; Astrea, G.; Barghigiani, M.; Battini, R.; Battisti, C.; Caputi, C.; Cioffi, E.; De Michele, G.; Dotti, M. T.; Fico, T.; Fiorillo, C.; Galosi, S.; Lieto, M.; Malandrini, A.; Melone, M. A. B.; Mignarri, A.; Natale, G.; Pegoraro, E.; Petrucci, A.; Ricca, I.; Riso, V.; Rossi, S.; Rubegni, A.; Scarlatti, A.; Tinelli, F.; Trovato, R.; Tedeschi, G.; Tessa, A.; Filla, A.; Santorelli, F. M., NGS in Hereditary Ataxia: When Rare Becomes Frequent. *Int J Mol Sci* **2021,** 22, (16).
126. Guo, H.; Wang, T.; Wu, H.; Long, M.; Coe, B. P.; Li, H.; Xun, G.; Ou, J.; Chen, B.; Duan, G.; Bai, T.; Zhao, N.; Shen, Y.; Li, Y.; Wang, Y.; Zhang, Y.; Baker, C.; Liu, Y.; Pang, N.; Huang, L.; Han, L.; Jia, X.; Liu, C.; Ni, H.; Yang, X.; Xia, L.; Chen, J.; Shen, L.; Li, Y.; Zhao, R.; Zhao, W.; Peng, J.; Pan, Q.; Long, Z.; Su, W.; Tan, J.; Du, X.; Ke, X.; Yao, M.; Hu, Z.; Zou, X.; Zhao, J.; Bernier, R. A.; Eichler, E. E.; Xia, K., Inherited and multiple de novo mutations in autism/developmental delay risk genes suggest a multifactorial model. *Mol Autism* **2018,** 9, 64.
127. Takata, A.; Miyake, N.; Tsurusaki, Y.; Fukai, R.; Miyatake, S.; Koshimizu, E.; Kushima, I.; Okada, T.; Morikawa, M.; Uno, Y.; Ishizuka, K.; Nakamura, K.; Tsujii, M.; Yoshikawa, T.; Toyota, T.; Okamoto, N.; Hiraki, Y.; Hashimoto, R.; Yasuda, Y.; Saitoh, S.; Ohashi, K.; Sakai, Y.; Ohga, S.; Hara, T.; Kato, M.; Nakamura, K.; Ito, A.; Seiwa, C.; Shirahata, E.; Osaka, H.; Matsumoto, A.; Takeshita, S.; Tohyama, J.; Saikusa, T.; Matsuishi, T.; Nakamura, T.; Tsuboi, T.; Kato, T.; Suzuki, T.; Saitsu, H.; Nakashima, M.; Mizuguchi, T.; Tanaka, F.; Mori, N.; Ozaki, N.; Matsumoto, N., Integrative Analyses of De Novo Mutations Provide Deeper Biological Insights into Autism Spectrum Disorder. *Cell Rep* **2018,** 22, (3), 734-747.
128. Tsoi, H.; Yu, A. C.; Chen, Z. S.; Ng, N. K.; Chan, A. Y.; Yuen, L. Y.; Abrigo, J. M.; Tsang, S. Y.; Tsui, S. K.; Tong, T. M.; Lo, I. F.; Lam, S. T.; Mok, V. C.; Wong, L. K.; Ngo, J. C.; Lau, K. F.; Chan, T. F.; Chan, H. Y., A novel missense mutation in CCDC88C activates the JNK pathway and causes a dominant form of spinocerebellar ataxia. *J Med Genet* **2014,** 51, (9), 590-5.
129. Yuen, R. K.; Merico, D.; Cao, H.; Pellecchia, G.; Alipanahi, B.; Thiruvahindrapuram, B.; Tong, X.; Sun, Y.; Cao, D.; Zhang, T.; Wu, X.; Jin, X.; Zhou, Z.; Liu, X.; Nalpathamkalam, T.; Walker, S.; Howe, J. L.; Wang, Z.; MacDonald, J. R.; Chan, A.; D'Abate, L.; Deneault, E.; Siu, M. T.; Tammimies, K.; Uddin, M.; Zarrei, M.; Wang, M.; Li, Y.; Wang, J.; Wang, J.; Yang, H.; Bookman, M.; Bingham, J.; Gross, S. S.; Loy, D.; Pletcher, M.; Marshall, C. R.; Anagnostou, E.; Zwaigenbaum, L.; Weksberg, R.; Fernandez, B. A.; Roberts, W.; Szatmari, P.; Glazer, D.; Frey, B. J.; Ring, R. H.; Xu, X.; Scherer, S. W., Genome-wide characteristics of de novo mutations in autism. *NPJ Genom Med* **2016,** 1, 160271-1602710.
130. Yahia, A.; Chen, Z. S.; Ahmed, A. E.; Emad, S.; Adil, R.; Abubaker, R.; Taha, S.; Salih, M. A.; Elsayed, L.; Chan, H. Y. E.; Stevanin, G., A heterozygous mutation in the CCDC88C gene likely causes early-onset pure hereditary spastic paraplegia: a case report. *BMC Neurol* **2021,** 21, (1), 78.
131. Hu, X.; Li, H.; Gui, B.; Xu, Y.; Wang, J.; Li, N.; Su, J.; Zhang, S.; Song, Y.; Wang, Y.; Luo, J.; Fan, X.; Wang, J.; Chen, S.; Gong, C.; Shen, Y., Prenatal and early diagnosis of Chinese 3-M syndrome patients with novel pathogenic variants. *Clin Chim Acta* **2017,** 474, 159-164.
132. Yang, L. L.; Liang, S. S., Study on pathogenic genes of dwarfism disease by next-generation sequencing. *World J Clin Cases* **2021,** 9, (7), 1600-1609.
133. Huang, Z.; Sun, Y.; Fan, Y.; Wang, L.; Liu, H.; Gong, Z.; Wang, J.; Yan, H.; Wang, Y.; Hu, G.; Wang, R.; Ye, J.; Han, L.; Qiu, W.; Zhang, H.; Liang, L.; Yang, Y.; Dauber, A.; Yu, Y.; Gu, X. F., Genetic Evaluation of 114 Chinese Short Stature Children in the Next Generation Era: a Single Center Study. *Cell Physiol Biochem* **2018,** 49, (1), 295-305.
134. Neale, B. M.; Kou, Y.; Liu, L.; Ma'ayan, A.; Samocha, K. E.; Sabo, A.; Lin, C. F.; Stevens, C.; Wang, L. S.; Makarov, V.; Polak, P.; Yoon, S.; Maguire, J.; Crawford, E. L.; Campbell, N. G.; Geller, E. T.; Valladares, O.; Schafer, C.; Liu, H.; Zhao, T.; Cai, G.; Lihm, J.; Dannenfelser, R.; Jabado, O.; Peralta, Z.; Nagaswamy, U.; Muzny, D.; Reid, J. G.; Newsham, I.; Wu, Y.; Lewis, L.; Han, Y.; Voight, B. F.; Lim, E.; Rossin, E.; Kirby, A.; Flannick, J.; Fromer, M.; Shakir, K.; Fennell, T.; Garimella, K.; Banks, E.; Poplin, R.; Gabriel, S.; DePristo, M.; Wimbish, J. R.; Boone, B. E.; Levy, S. E.; Betancur, C.; Sunyaev, S.; Boerwinkle, E.; Buxbaum, J. D.; Cook, E. H., Jr.; Devlin, B.; Gibbs, R. A.; Roeder, K.; Schellenberg, G. D.; Sutcliffe, J. S.; Daly, M. J., Patterns and rates of exonic de novo mutations in autism spectrum disorders. *Nature* **2012,** 485, (7397), 242-5.

135. Nishioka, M.; Kazuno, A. A.; Nakamura, T.; Sakai, N.; Hayama, T.; Fujii, K.; Matsuo, K.; Komori, A.; Ishiwata, M.; Watanabe, Y.; Oka, T.; Matoba, N.; Kataoka, M.; Alkanaq, A. N.; Hamanaka, K.; Tsuboi, T.; Sengoku, T.; Ogata, K.; Iwata, N.; Ikeda, M.; Matsumoto, N.; Kato, T.; Takata, A., Systematic analysis of exonic germline and postzygotic de novo mutations in bipolar disorder. *Nat Commun* **2021,** 12, (1), 3750.
136. Cappi, C.; Oliphant, M. E.; Peter, Z.; Zai, G.; Conceicao do Rosario, M.; Sullivan, C. A. W.; Gupta, A. R.; Hoffman, E. J.; Virdee, M.; Olfson, E.; Abdallah, S. B.; Willsey, A. J.; Shavitt, R. G.; Miguel, E. C.; Kennedy, J. L.; Richter, M. A.; Fernandez, T. V., De Novo Damaging DNA Coding Mutations Are Associated With Obsessive-Compulsive Disorder and Overlap With Tourette's Disorder and Autism. *Biol Psychiatry* **2020,** 87, (12), 1035-1044.
137. Higa, L. A.; Wardley, J.; Wardley, C.; Singh, S.; Foster, T.; Shen, J. J., CNKSR2-related neurodevelopmental and epilepsy disorder: a cohort of 13 new families and literature review indicating a predominance of loss of function pathogenic variants. *BMC Med Genomics* **2021,** 14, (1), 186.
138. Stranneheim, H.; Lagerstedt-Robinson, K.; Magnusson, M.; Kvarnung, M.; Nilsson, D.; Lesko, N.; Engvall, M.; Anderlid, B. M.; Arnell, H.; Johansson, C. B.; Barbaro, M.; Bjorck, E.; Bruhn, H.; Eisfeldt, J.; Freyer, C.; Grigelioniene, G.; Gustavsson, P.; Hammarsjo, A.; Hellstrom-Pigg, M.; Iwarsson, E.; Jemt, A.; Laaksonen, M.; Enoksson, S. L.; Malmgren, H.; Naess, K.; Nordenskjold, M.; Oscarson, M.; Pettersson, M.; Rasi, C.; Rosenbaum, A.; Sahlin, E.; Sardh, E.; Stodberg, T.; Tesi, B.; Tham, E.; Thonberg, H.; Tohonen, V.; von Dobeln, U.; Vassiliou, D.; Vonlanthen, S.; Wikstrom, A. C.; Wincent, J.; Winqvist, O.; Wredenberg, A.; Ygberg, S.; Zetterstrom, R. H.; Marits, P.; Soller, M. J.; Nordgren, A.; Wirta, V.; Lindstrand, A.; Wedell, A., Integration of whole genome sequencing into a healthcare setting: high diagnostic rates across multiple clinical entities in 3219 rare disease patients. *Genome Med* **2021,** 13, (1), 40.
139. Bonardi, C. M.; Mignot, C.; Serratosa, J. M.; Giraldez, B. G.; Moretti, R.; Rudolf, G.; Reale, C.; Gellert, P. M.; Johannesen, K. M.; Lesca, G.; Tassinari, C. A.; Gardella, E.; Moller, R. S.; Rubboli, G., Expanding the clinical and EEG spectrum of CNKSR2-related encephalopathy with status epilepticus during slow sleep (ESES). *Clin Neurophysiol* **2020,** 131, (5), 1030-1039.
140. Vaags, A. K.; Bowdin, S.; Smith, M. L.; Gilbert-Dussardier, B.; Brocke-Holmefjord, K. S.; Sinopoli, K.; Gilles, C.; Haaland, T. B.; Vincent-Delorme, C.; Lagrue, E.; Harbuz, R.; Walker, S.; Marshall, C. R.; Houge, G.; Kalscheuer, V. M.; Scherer, S. W.; Minassian, B. A., Absent CNKSR2 causes seizures and intellectual, attention, and language deficits. *Ann Neurol* **2014,** 76, (5), 758-64.
141. Toraman, B.; Bilginer, S. C.; Hesapcioglu, S. T.; Goker, Z.; Soykam, H. O.; Erguner, B.; Dincer, T.; Yildiz, G.; Unsal, S.; Kasap, B. K.; Kandil, S.; Kalay, E., Finding underlying genetic mechanisms of two patients with autism spectrum disorder carrying familial apparently balanced chromosomal translocations. *J Gene Med* **2021,** 23, (4), e3322.
142. Costain, G.; Cordeiro, D.; Matviychuk, D.; Mercimek-Andrews, S., Clinical Application of Targeted Next-Generation Sequencing Panels and Whole Exome Sequencing in Childhood Epilepsy. *Neuroscience* **2019,** 418, 291-310.
143. Daoqi, M.; Guohong, C.; Yuan, W.; Zhixiao, Y.; Kaili, X.; Shiyue, M., Exons deletion of CNKSR2 gene identified in X-linked syndromic intellectual disability. *BMC Med Genet* **2020,** 21, (1), 69.
144. Zhang, Y.; Yu, T.; Li, N.; Wang, J.; Wang, J.; Ge, Y.; Yao, R., Psychomotor development and attention problems caused by a splicing variant of CNKSR2. *BMC Med Genomics* **2020,** 13, (1), 182.
145. Polla, D. L.; Saunders, H. R.; de Vries, B. B. A.; van Bokhoven, H.; de Brouwer, A. P. M., A de novo variant in the X-linked gene CNKSR2 is associated with seizures and mild intellectual disability in a female patient. *Mol Genet Genomic Med* **2019,** 7, (10), e00861.
146. Iuso, A.; Alhaddad, B.; Weigel, C.; Kotzaeridou, U.; Mastantuono, E.; Schwarzmayr, T.; Graf, E.; Terrile, C.; Prokisch, H.; Strom, T. M.; Hoffmann, G. F.; Meitinger, T.; Haack, T. B., A Homozygous Splice Site Mutation in SLC25A42, Encoding the Mitochondrial Transporter of Coenzyme A, Causes Metabolic Crises and Epileptic Encephalopathy. *JIMD Rep* **2019,** 44, 1-7.
147. Aldosary, M.; Baselm, S.; Abdulrahim, M.; Almass, R.; Alsagob, M.; AlMasseri, Z.; Huma, R.; AlQuait, L.; Al-Shidi, T.; Al-Obeid, E.; AlBakheet, A.; Alahideb, B.; Alahaidib, L.; Qari, A.; Taylor, R. W.; Colak, D.; AlSayed, M. D.; Kaya, N., SLC25A42-associated mitochondrial encephalomyopathy: Report of additional founder cases and functional characterization of a novel deletion. *JIMD Rep* **2021,** 60, (1), 75-87.

148. Almannai, M.; Alasmari, A.; Alqasmi, A.; Faqeih, E.; Al Mutairi, F.; Alotaibi, M.; Samman, M. M.; Eyaid, W.; Aljadhai, Y. I.; Shamseldin, H. E.; Craigen, W.; Alkuraya, F. S., Expanding the phenotype of SLC25A42-associated mitochondrial encephalomyopathy. *Clin Genet* **2018,** 93, (5), 1097-1102.
149. Bertoli-Avella, A. M.; Beetz, C.; Ameziane, N.; Rocha, M. E.; Guatibonza, P.; Pereira, C.; Calvo, M.; Herrera-Ordonez, N.; Segura-Castel, M.; Diego-Alvarez, D.; Zawada, M.; Kandaswamy, K. K.; Werber, M.; Paknia, O.; Zielske, S.; Ugrinovski, D.; Warnack, G.; Kampe, K.; Iurascu, M. I.; Cozma, C.; Vogel, F.; Alhashem, A.; Hertecant, J.; Al-Shamsi, A. M.; Alswaid, A. F.; Eyaid, W.; Al Mutairi, F.; Alfares, A.; Albalwi, M. A.; Alfadhel, M.; Al-Sannaa, N. A.; Reardon, W.; Alanay, Y.; Rolfs, A.; Bauer, P., Successful application of genome sequencing in a diagnostic setting: 1007 index cases from a clinically heterogeneous cohort. *Eur J Hum Genet* **2021,** 29, (1), 141-153.
150. Shamseldin, H. E.; Smith, L. L.; Kentab, A.; Alkhalidi, H.; Summers, B.; Alsedairy, H.; Xiong, Y.; Gupta, V. A.; Alkuraya, F. S., Mutation of the mitochondrial carrier SLC25A42 causes a novel form of mitochondrial myopathy in humans. *Hum Genet* **2016,** 135, (1), 21-30.
151. Vorstman, J. A. S.; Olde Loohuis, L. M.; Investigators, G.; Kahn, R. S.; Ophoff, R. A.; investigators, G., Double hits in schizophrenia. *Hum Mol Genet* **2018,** 27, (15), 2755-2761.
152. Kushima, I.; Aleksic, B.; Nakatochi, M.; Shimamura, T.; Shiino, T.; Yoshimi, A.; Kimura, H.; Takasaki, Y.; Wang, C.; Xing, J.; Ishizuka, K.; Oya-Ito, T.; Nakamura, Y.; Arioka, Y.; Maeda, T.; Yamamoto, M.; Yoshida, M.; Noma, H.; Hamada, S.; Morikawa, M.; Uno, Y.; Okada, T.; Iidaka, T.; Iritani, S.; Yamamoto, T.; Miyashita, M.; Kobori, A.; Arai, M.; Itokawa, M.; Cheng, M. C.; Chuang, Y. A.; Chen, C. H.; Suzuki, M.; Takahashi, T.; Hashimoto, R.; Yamamori, H.; Yasuda, Y.; Watanabe, Y.; Nunokawa, A.; Someya, T.; Ikeda, M.; Toyota, T.; Yoshikawa, T.; Numata, S.; Ohmori, T.; Kunimoto, S.; Mori, D.; Iwata, N.; Ozaki, N., High-resolution copy number variation analysis of schizophrenia in Japan. *Mol Psychiatry* **2017,** 22, (3), 430-440.
153. Khan, F. F.; Melton, P. E.; McCarthy, N. S.; Morar, B.; Blangero, J.; Moses, E. K.; Jablensky, A., Whole genome sequencing of 91 multiplex schizophrenia families reveals increased burden of rare, exonic copy number variation in schizophrenia probands and genetic heterogeneity. *Schizophr Res* **2018,** 197, 337-345.
154. Kushima, I.; Aleksic, B.; Nakatochi, M.; Shimamura, T.; Okada, T.; Uno, Y.; Morikawa, M.; Ishizuka, K.; Shiino, T.; Kimura, H.; Arioka, Y.; Yoshimi, A.; Takasaki, Y.; Yu, Y.; Nakamura, Y.; Yamamoto, M.; Iidaka, T.; Iritani, S.; Inada, T.; Ogawa, N.; Shishido, E.; Torii, Y.; Kawano, N.; Omura, Y.; Yoshikawa, T.; Uchiyama, T.; Yamamoto, T.; Ikeda, M.; Hashimoto, R.; Yamamori, H.; Yasuda, Y.; Someya, T.; Watanabe, Y.; Egawa, J.; Nunokawa, A.; Itokawa, M.; Arai, M.; Miyashita, M.; Kobori, A.; Suzuki, M.; Takahashi, T.; Usami, M.; Kodaira, M.; Watanabe, K.; Sasaki, T.; Kuwabara, H.; Tochigi, M.; Nishimura, F.; Yamasue, H.; Eriguchi, Y.; Benner, S.; Kojima, M.; Yassin, W.; Munesue, T.; Yokoyama, S.; Kimura, R.; Funabiki, Y.; Kosaka, H.; Ishitobi, M.; Ohmori, T.; Numata, S.; Yoshikawa, T.; Toyota, T.; Yamakawa, K.; Suzuki, T.; Inoue, Y.; Nakaoka, K.; Goto, Y. I.; Inagaki, M.; Hashimoto, N.; Kusumi, I.; Son, S.; Murai, T.; Ikegame, T.; Okada, N.; Kasai, K.; Kunimoto, S.; Mori, D.; Iwata, N.; Ozaki, N., Comparative Analyses of Copy-Number Variation in Autism Spectrum Disorder and Schizophrenia Reveal Etiological Overlap and Biological Insights. *Cell Rep* **2018,** 24, (11), 2838-2856.
155. Cukier, H. N.; Dueker, N. D.; Slifer, S. H.; Lee, J. M.; Whitehead, P. L.; Lalanne, E.; Leyva, N.; Konidari, I.; Gentry, R. C.; Hulme, W. F.; Booven, D. V.; Mayo, V.; Hofmann, N. K.; Schmidt, M. A.; Martin, E. R.; Haines, J. L.; Cuccaro, M. L.; Gilbert, J. R.; Pericak-Vance, M. A., Exome sequencing of extended families with autism reveals genes shared across neurodevelopmental and neuropsychiatric disorders. *Mol Autism* **2014,** 5, (1), 1.
156. Ruiz-Martinez, J.; Azcona, L. J.; Bergareche, A.; Marti-Masso, J. F.; Paisan-Ruiz, C., Whole-exome sequencing associates novel CSMD1 gene mutations with familial Parkinson disease. *Neurol Genet* **2017,** 3, (5), e177.
157. Bradley, W. E.; Raelson, J. V.; Dubois, D. Y.; Godin, E.; Fournier, H.; Prive, C.; Allard, R.; Pinchuk, V.; Lapalme, M.; Paulussen, R. J.; Belouchi, A., Hotspots of large rare deletions in the human genome. *PLoS One* **2010,** 5, (2), e9401.
158. Sanders, S. J.; Murtha, M. T.; Gupta, A. R.; Murdoch, J. D.; Raubeson, M. J.; Willsey, A. J.; Ercan-Sencicek, A. G.; DiLullo, N. M.; Parikshak, N. N.; Stein, J. L.; Walker, M. F.; Ober, G. T.; Teran, N. A.; Song, Y.; El-Fishawy, P.; Murtha, R. C.; Choi, M.; Overton, J. D.; Bjornson, R. D.; Carriero, N. J.; Meyer, K. A.; Bilguvar, K.; Mane, S. M.; Sestan, N.; Lifton, R. P.; Gunel, M.; Roeder, K.; Geschwind, D. H.; Devlin, B.; State, M. W., De novo mutations revealed by whole-exome sequencing are strongly associated with autism. *Nature* **2012,** 485, (7397), 237-41.

159. Gambin, T.; Yuan, B.; Bi, W.; Liu, P.; Rosenfeld, J. A.; Coban-Akdemir, Z.; Pursley, A. N.; Nagamani, S. C. S.; Marom, R.; Golla, S.; Dengle, L.; Petrie, H. G.; Matalon, R.; Emrick, L.; Proud, M. B.; Treadwell-Deering, D.; Chao, H. T.; Koillinen, H.; Brown, C.; Urraca, N.; Mostafavi, R.; Bernes, S.; Roeder, E. R.; Nugent, K. M.; Bader, P. I.; Bellus, G.; Cummings, M.; Northrup, H.; Ashfaq, M.; Westman, R.; Wildin, R.; Beck, A. E.; Immken, L.; Elton, L.; Varghese, S.; Buchanan, E.; Faivre, L.; Lefebvre, M.; Schaaf, C. P.; Walkiewicz, M.; Yang, Y.; Kang, S. L.; Lalani, S. R.; Bacino, C. A.; Beaudet, A. L.; Breman, A. M.; Smith, J. L.; Cheung, S. W.; Lupski, J. R.; Patel, A.; Shaw, C. A.; Stankiewicz, P., Identification of novel candidate disease genes from de novo exonic copy number variants. *Genome Med* **2017,** 9, (1), 83.

160. Saleh, S.; Beyyumi, E.; Al Kaabi, A.; Hertecant, J.; Barakat, D.; Al Dhaheri, N. S.; Al-Gazali, L.; Al Shamsi, A., Spectrum of neuro-genetic disorders in the United Arab Emirates national population. *Clin Genet* **2021,** 100, (5), 573-600.

161. Lahbib, S.; Trabelsi, M.; Dallali, H.; Sakka, R.; Bourourou, R.; Kefi, R.; Mrad, R.; Abdelhak, S.; Gaddour, N., Novel MED12 variant in a multiplex Fragile X syndrome family: dual molecular etiology of two X-linked intellectual disabilities with autism in the same family. *Mol Biol Rep* **2019,** 46, (4), 4185-4193.

162. Schizophrenia Psychiatric Genome-Wide Association Study, C., Genome-wide association study identifies five new schizophrenia loci. *Nat Genet* **2011,** 43, (10), 969-76.

163. Yu, Y.; Lin, Y.; Takasaki, Y.; Wang, C.; Kimura, H.; Xing, J.; Ishizuka, K.; Toyama, M.; Kushima, I.; Mori, D.; Arioka, Y.; Uno, Y.; Shiino, T.; Nakamura, Y.; Okada, T.; Morikawa, M.; Ikeda, M.; Iwata, N.; Okahisa, Y.; Takaki, M.; Sakamoto, S.; Someya, T.; Egawa, J.; Usami, M.; Kodaira, M.; Yoshimi, A.; Oya-Ito, T.; Aleksic, B.; Ohno, K.; Ozaki, N., Rare loss of function mutations in N-methyl-D-aspartate glutamate receptors and their contributions to schizophrenia susceptibility. *Transl Psychiatry* **2018,** 8, (1), 12.

164. Hornig, T.; Gruning, B.; Kundu, K.; Houwaart, T.; Backofen, R.; Biber, K.; Normann, C., GRIN3B missense mutation as an inherited risk factor for schizophrenia: whole-exome sequencing in a family with a familiar history of psychotic disorders. *Genet Res (Camb)* **2017,** 99, e1.

165. Matsuno, H.; Ohi, K.; Hashimoto, R.; Yamamori, H.; Yasuda, Y.; Fujimoto, M.; Yano-Umeda, S.; Saneyoshi, T.; Takeda, M.; Hayashi, Y., A naturally occurring null variant of the NMDA type glutamate receptor NR3B subunit is a risk factor of schizophrenia. *PLoS One* **2015,** 10, (3), e0116319.
